# The chromosome-scale genome and the genetic resistance machinery against insect herbivores of the Mexican toloache, *Datura stramonium*

**DOI:** 10.1093/g3journal/jkad288

**Published:** 2023-12-19

**Authors:** Ivan M De-la-Cruz, Ken Oyama, Juan Núñez-Farfán

**Affiliations:** Departamento de Ecología Evolutiva, Instituto de Ecología, Universidad Nacional Autónoma de México, Mexico City 04510, Mexico; Department of Plant Protection Biology, Swedish University of Agricultural Sciences, Lomma, Alnarp 230 53, Sweden; Escuela Nacional de Estudios Superiores (ENES), Universidad Nacional Autónoma de México (UNAM), Campus Morelia, Morelia, Michoacán 8701, Mexico; Departamento de Ecología Evolutiva, Instituto de Ecología, Universidad Nacional Autónoma de México, Mexico City 04510, Mexico

**Keywords:** genomics of plant defense, herbivory, plant resistance, Solanaceae, quantitative trait loci (QTLs) of plant resistance, Plant Genetics and Genomics

## Abstract

Plant resistance refers to the heritable ability of plants to reduce damage caused by natural enemies, such as herbivores and pathogens, either through constitutive or induced traits like chemical compounds or trichomes. However, the genetic architecture—the number and genome location of genes that affect plant defense and the magnitude of their effects—of plant resistance to arthropod herbivores in natural populations remains poorly understood. In this study, we aimed to unveil the genetic architecture of plant resistance to insect herbivores in the annual herb *Datura stramonium* (Solanaceae) through quantitative trait loci mapping. We achieved this by assembling the species’ genome and constructing a linkage map using an F_2_ progeny transplanted into natural habitats. Furthermore, we conducted differential gene expression analysis between undamaged and damaged plants caused by the primary folivore, *Lema daturaphila* larvae. Our genome assembly resulted in 6,109 scaffolds distributed across 12 haploid chromosomes. A single quantitative trait loci region on chromosome 3 was associated with plant resistance, spanning 0 to 5.17 cM. The explained variance by the quantitative trait loci was 8.44%. Our findings imply that the resistance mechanisms of *D. stramonium* are shaped by the complex interplay of multiple genes with minor effects. Protein–protein interaction networks involving genes within the quantitative trait loci region and overexpressed genes uncovered the key role of receptor-like cytoplasmic kinases in signaling and regulating tropane alkaloids and terpenoids, which serve as powerful chemical defenses against *D. stramonium* herbivores. The data generated in our study constitute important resources for delving into the evolution and ecology of secondary compounds mediating plant–insect interactions.

## Introduction

Plants have evolved a myriad of defense mechanisms to resist natural enemies. These defensive characteristics function as both protective and antagonistic mechanisms against herbivores, pathogens, and competitors (Levin 1973; Kessler and Baldwin 2001; Agrawal and Fishbein 2006; Runyon *et al.* 2010; Stenberg and Muola 2017). Defensive traits encompass morphological features, such as trichomes and spines, as well as chemical compounds (War *et al.* 2012; Mitchell *et al.* 2016). Less conspicuous plant defense characters involve structures like extrafloral nectaries and the release of volatile organic compounds (VOCs) to indirectly attract predators of their adversaries, often referred to as “bodyguards” (Kessler and Baldwin 2001, 2002; War *et al.* 2012). The evolution of such diverse resistance traits in plants is believed to be influenced by variations in local selection pressures, differences in the type and community of herbivores/pathogens that plants face, and on extant genetic variation and the genetic architecture of traits. Consequently, this has led to either shared or distinct genetic pathways for defending against enemies (Ehrlich and Raven 1964; Rausher 2001; Futuyma and Agrawal 2009; Maron *et al.* 2019). Nonetheless, deciphering the genetic mechanisms underlying these resistance traits remains challenging, primarily because of their polygenic nature (Young 1996; Corwin and Kliebenstein 2017). Moreover, most of our understanding of genes underpinning plant resistance originates from plant–pathogen research in model systems. Few investigations have explored the genetic architecture of plant resistance to specialist herbivores in natural populations (Kliebenstein *et al.* 2002; Weinig *et al.* 2003; Pfalz *et al.* 2009; Meihls *et al.* 2012).

Genes responsible for plant resistance have been naturally present in plant populations and were harnessed in early phases of crop domestication (Kourelis and van der Hoorn 2018). However, throughout the 20th century, the focus on the genetics of plant herbivore resistance was largely overshadowed by the development of inexpensive chemical pesticides (Stenberg 2017). Direct defense traits often faced negative selection pressures because they could compromise crop yield, flavor, and texture (Stenberg 2017). Meanwhile, the significance of indirect defense mechanisms was generally neglected due to a lack of awareness of their ecological relevance (Stenberg *et al.* 2015). Although modern cultivars have largely lost both direct and indirect defense traits, recent studies indicate that these crucial traits are preserved in wild relatives of crop species (Hajjar and Hodgkin 2007; Muola *et al.* 2017). Thus, one of the fundamental challenges for contemporary plant breeders is to identify and incorporate this genetic variability from wild crop relatives to bolster the innate resistance of future cultivars against pests and pathogens (Hajjar and Hodgkin 2007; Stenberg 2017). Achieving this objective demands an in-depth understanding of the genetic architecture that controls plant defense mechanisms and modulates interactions with herbivores (quantitative resistance loci; Corwin and Kliebenstein 2017). Such insights not only contribute to the evolutionary biology of plant defense but also empower breeders to introduce allelic variants with the potential to enhance crop resilience in light of changing environments (Stenberg and Ortiz 2021).

**Fig. 1.**
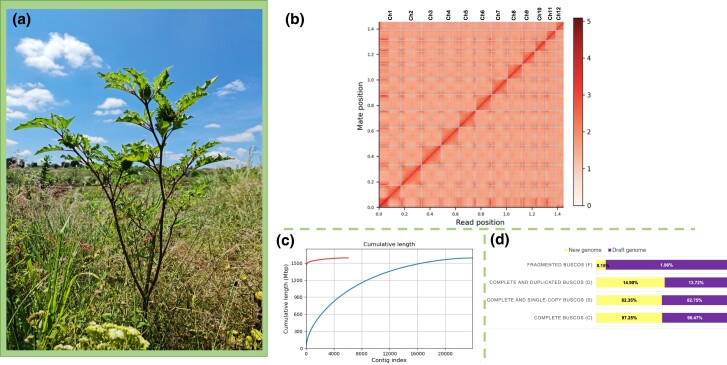
a) *Datura stramonium* is part of the Solanaceae family, and Mexico is considered its center of origin. This species grows in ruderal and urban areas and nowadays is distributed worldwide. In Mexico, it is attacked mainly by 3 main herbivores: the beetles *L. daturaphila* (folivore), *T. soror* (seed predator), and *E. parvula* (folivore). It is well known due to its production of tropane alkaloids which are used in traditional medicine and pharmaceutical industry. b) Linkage density histogram of the Omni-C sequences. In this figure, the x- and y-axes give the mapping positions of the first and second reads in the read pair, respectively, grouped into bins. The color of each square gives the number of read pairs within that bin. White vertical and black horizontal lines have been added to show the borders between scaffolds. Scaffolds less than 1 Mb were excluded. c) Cumulative length plot displaying the growth of contig lengths for the draft (blue line) and improved (red lines) genome of *D. stramonium*. On the x-axis, contigs are ordered from the largest to smallest. The y-axis gives the size of the *x* largest contigs in the assembly. d) BUSCO plots for the improved *D. stramonium* genome. The plot shows quantitative measures for the assessment of the genome completeness based on evolutionarily informed expectations of gene content from near-universal single-copy orthologs selected from the “Solanaceae odb10*” database. Credit for Fig. 1a to Juan Núñez-Farfán. See Table 1.

The Solanaceae herb, *Datura stramonium*, known as “toloache” in Mexico (Fig. 1a), has become widely distributed across the globe and has its origins in Mexico (Geeta and Gharaibeh 2007). This annual herb is renowned for its tropane alkaloids and terpenoids, substances utilized in both pre-Columbian traditional medicine (De la Cruz 1552) and modern pharmaceuticals (Jirschitzka *et al.* 2012; Kohnen-Johannsen and Kayser 2019). While tropane alkaloids are found across multiple Solanaceae species (Huang *et al.* 2021), limited research has investigated their ecological function as potential chemical defenses against pathogens and herbivores, with notable exceptions in the case of *D. stramonium* (Shonle and Bergelson 2000; Castillo *et al.* 2013, 2014; Miranda-Peréz *et al.* 2016; De-la-Cruz, Cruz, *et al.* 2020; De-la-Cruz, Merilä, *et al.* 2020). Prior ecological genetics studies focusing on defense mechanisms of *D. stramonium* against herbivores highlighted the genomic analysis of candidate genes (De-la-Cruz *et al.* 2021, 2022). Our findings also revealed that resistance in *D. stramonium* is both inheritable and subject to positive selection (De-la-Cruz, Merilä, *et al.* 2020). In this study, our primary objective was to pinpoint the quantitative trait loci (QTLs) associated with the plant’s resistance to its principal insect herbivores. To achieve this, we generated F_2_ progeny by crossing 2 distinct parental plants from different populations of the annual herb *D. stramonium* (Solanaceae). These populations exhibit variations in chemical defense levels and herbivore communities (De-la-Cruz, Cruz, *et al.* 2020, De-la-Cruz, Merilä, *et al.* 2020). These F_2_ progeny were transplanted into 2 distinct experimental sites situated within the native populations of the parents in Mexico, under natural conditions (De-la-Cruz, Merilä, *et al.* 2020). In addition, we improved the genome assembly of a *D. stramonium* specimen that is native to Mexico, achieving resolution at the chromosomal level. Utilizing this reference genome, we constructed a linkage map for our QTL analysis. To further elucidate the genetic mechanisms controlling plant resistance in this species, we performed a differential gene expression analysis (DGEA). We compared gene expression profiles between plants that were undamaged and those that had been subjected to feeding by *Lema daturaphila*, the primary chewing herbivore known to target this species.

## Methods

### Experimental design

An F_2_ generation progeny of *D. stramonium* was generated (Fig. 1a) through a cross between 2 grandparents with differing levels of resistance to herbivores. The pollen donor originated from Ticumán, while the pollen receptor plant was from Teotihuacán (De-la-Cruz, Cruz, *et al.* 2020; De-la-Cruz, Merilä, *et al.* 2020). Previous investigations have shown that plants from Ticumán exhibit higher resistance (1 − % of leaf area consumed = 0.89 ± 0.004) to herbivores in comparison with plants from Teotihuacán (1 − % of leaf area consumed = 0.63 ± 0.029) (Valverde *et al.* 2001, 2003; Fornoni *et al.* 2003; Castillo *et al.* 2013). Additional information regarding the complete experimental design can be found in De-la-Cruz, Merilä, *et al.* (2020).

Briefly, for the production of the F_1_ and F_2_ progeny employed in this study, we initially identified and analyzed a total of 21 tropane alkaloids in the parental plants. These parental plants consisted of 45 and 47 distinct individuals from Teotihuacán and Ticumán, respectively. The analytical methods used for this study are described in De-la-Cruz, Cruz, *et al.* (2020). The cumulative concentration of all alkaloids determined the overall tropane alkaloid concentration for each parental plant. Subsequently, we selected the pair of parental plants that exhibited the most significant difference in total tropane alkaloid concentration: Teotihuacán 1 and Ticumán 23. These specific plants displayed a 58-times higher contrast in their total alkaloid concentration (1,013 ng/g of leaf for Teotihuacán 1 and 59,000 ng/g of leaf for Ticumán 23, respectively).

Germinated F_1_ seeds resulting from the cross between these 2 selected parental plants were cultivated and grown following the procedures outlined in De-la-Cruz, Cruz, *et al.* (2020) and De-la-Cruz, Merilä, *et al.* (2020). To initiate the germination of the F_1_ progeny, we utilized seeds from 3 fruits resulting from the same selected cross between Teotihuacán 1 and Ticumán 23. From among the germinated F_1_ plants (*n* = 8), we randomly chose a single plant, and its flowers were carefully enclosed in bags to prevent any pollen contamination from other plants, even though all the plants were cultivated in a controlled glasshouse environment. This particular F_1_ individual was allowed to undergo self-pollination to generate the F_2_ generation progeny, forming a single-family lineage. The seeds from the parental plants, F_1_, and F_2_ progenies were germinated by immersing them in water containers and maintaining them within an environmental chamber under a photoperiod of 12:12 L:D, at a temperature of 30°C during the day and 25°C at night, with a constant humidity of 85%. To promote germination, the seeds were scarified. Subsequently, the germinated F_2_ seeds were transplanted into plastic pots (237 mL) filled with a 1:1 mixture of sand and vermiculite and then randomly distributed across benches within the greenhouse. Each F_2_ plant received a uniform daily water supply (500 mL) until they were relocated to natural conditions.

### Experiment (plant damage)

#### Experiment

F_2_ seeds, taken randomly, were germinated and grown in the greenhouse as described above. When the 2 true leaves appeared, F_2_ seedlings (*n* = 230) were transplanted to an experimental plot in Teotihuacán and Ticumán to expose the F_2_ plants to the local herbivores and natural environmental conditions of the grandparents (De-la-Cruz, Merilä, *et al.* 2020). In the study site, seedlings were planted in the experimental plot according to a complete randomized design. Plants were spaced 1 m apart in a regular grid. Experimental plot was regularly weeded to prevent interference and competition by other plant species.

#### Damage by herbivores

Percent of leaf area consumed by chewing herbivores was measured with the mobile application BioLeaf (Machado *et al.* 2016) on 4 sampling periods (15, 30, 45, 60 days after planting). On each sampling date, we took photographs of 8 randomly chosen full expanded leaves per plant using a mobile phone (Samsung Galaxy S6 edge). The app automatically calculates the injured leaf regions caused by insect herbivory and then estimates the damage (in percentage) relative to the total leaf area (Machado *et al.* 2016). Thus, we were able to quantify the average proportion of leaf damage by herbivores per plant among the 4 sampling periods which corresponds to the entire growing season of *D. stramonium* in each year. It is important to highlight that in the Teotihuacán site, most leaf tissue was completely eaten by herbivores in many plants. In these cases, we assigned 100% of the damage to these plants (De-la-Cruz, Cruz, *et al.* 2020). We used the inverse of plant damage as a measure of plant resistance (calculated using the operational definition 1 − mean proportion of leaf area damaged; Simms and Rausher 1987, 2001).

### Improving the draft genome assembly of *D. stramonium*

#### Scaffolding using long-read sequences and RNA-seq

We employed long-reads obtained through PacBio Sequel I sequencing (Pacific Biosciences) from the BioProject PRJNA622882 to enhance the previously published draft genome of *D. stramonium*, as described in De-la-Cruz *et al.* (2021). This process was carried out using LRScaf v1.1.10 (available at https://github.com/shingocat/lrscaf). Initially, a total of 9,995,713 PacBio reads were aligned to the draft genome using Minimap2 v2.24 (Li 2018). Subsequently, the alignment output file generated by Minimap2 was utilized as input for LRScaf. Additionally, we obtained 86,626,733 paired-end RNA sequences from 6 different individuals of *D. stramonium* as part of the BioProject PRJNA669339. These sequences were aligned to the draft genome produced by LRScaf using HISAT2 v2.2.1 (Kim *et al.* 2019). Following alignment, we obtained 6 “.bam” files, which were further sorted using SAMtools v1.8 (Li *et al.* 2009). These sorted “.bam” files were then employed as input for the Rascaf v1.0.2 program (Song *et al.* 2016). Rascaf utilizes continuity and order information derived from paired-end RNA-seq reads to enhance a draft assembly, with particular emphasis on improving gene regions (Song *et al.* 2016).

The new draft genome assembly was polished using the program Pilon (Walker *et al.* 2014). Polishing the contigs using this program brings the error rate down to 0.01% or lower (Chakraborty *et al.* 2019). First, we aligned the raw Illumina sequences, which were previously used for the assembly of the initial version of the *D. stramonium* draft genome (De-la-Cruz *et al.* 2021), to the newly generated genome assembly. Bowtie2 (Langmead and Salzberg 2012) was employed for this alignment step. Subsequently, we utilized SAMtools to transform, sort, and index the resulting alignment output. The polished draft genome was then generated by applying the Pilon program. This refined draft genome assembly served as the input for HiRise scaffolding, a software pipeline designed for leveraging proximity ligation data to scaffold genome assemblies (Putnam *et al.* 2016).

#### Omni-C library preparation and HiRise assembly

gDNA was extracted from 1 individual of *D. stramonium* from the locality of Ticumán, State of Morelos, Mexico (18°45′39.90″N, 99°7′13.86″W). gDNA was isolated from fresh leaves with a modified CTAB mini-prep protocol (Doyle 1991). The total amount of gDNA was measured using Qubit dsDNA HS Assay Kit (Invitrogen, Thermo Fisher Scientific, Waltham, USA). A total of 200 ng of gDNA were used for Omni-C library preparation. For Omni-C library preparation, chromatin was fixed in place with formaldehyde in the nucleus and then extracted (Putnam *et al.* 2016). Fixed chromatin was digested with DNAse I; chromatin ends were repaired and ligated to a biotinylated bridge adapter followed by proximity ligation of adapter containing ends (Putnam *et al.* 2016). After proximity ligation, crosslinks were reversed, and the DNA was purified. Purified DNA was treated to remove biotin that was not internal to ligated fragments (Putnam *et al.* 2016). Sequencing libraries were generated using NEBNext Ultra enzymes and Illumina-compatible adapters (Putnam *et al.* 2016). Biotin-containing fragments were isolated using streptavidin beads before PCR enrichment of each library (Putnam *et al.* 2016). The library was sequenced on an Illumina HiSeq X platform (Illumina Inc., San Diego, California, USA) to produce an ∼30× sequence coverage. Sequences with a quality score > 50 (FasQC; Andrews 2010) were kept for HiRise assembly.

The improved draft genome assembly, which resulted from the integration of long-read sequences and RNA-seq data (as previously described), along with Dovetail Omni-C library sequences, served as the primary input data for the HiRise assembler (Putnam *et al.* 2016). The Dovetail Omni-C library sequences were initially aligned to the draft input assembly using the bwa program v0.7.17 (Li and Durbin 2009). The separations of Dovetail Omni-C read pairs mapped within draft scaffolds were analyzed by HiRise to produce a likelihood model for genomic distance between read pairs, and the model was used to identify and break putative misjoins, score prospective joins, and make joins above a threshold. Omni-C library construction and HiRise scaffolding were done in the company Dovetail Genomics (Scotts Valley, California, USA).

#### Structural and functional genome annotation

Leaf and flower tissues were both obtained from the same individual utilized for DNA extraction for the subsequent preparation of the Omni-C library. Subsequently, RNA extraction was conducted by Dovetail Genomics. The resulting RNA was then subjected to paired-end sequencing on a HiSeq X platform (Illumina Inc., San Diego, California, USA). The genome annotation process proceeded as follows: Initially, repeat families present in the *D. stramonium* genome assembly were identified and classified de novo using the software package RepeatModeler v2.0.1 (Flynn *et al.* 2020). The custom repeat library generated from RepeatModeler was subsequently employed to detect, identify, and mask the repeats within the assembly file, using RepeatMasker v4.1.0 (Flynn *et al.* 2020). Coding sequences from *Arabidopsis thaliana*, *Capsicum annuum*, *Nicotiana attenuata*, and *Solanum lycopersicum* from EnsemblPlants were downloaded and used to train the initial ab initio model for *D. stramonium* using the AUGUSTUS software v2.5.5 (Stanke *et al.* 2006). Six rounds of prediction optimization were done with the scripts provided by AUGUSTUS. The same coding sequences were also used to train a separate ab initio model for *D. stramonium* using SNAP v2006.07.28 (Korf 2004). RNA sequences of *D. stramonium* generated in this work (see above) were also mapped onto the reference genome using the STAR aligner software v2.7 (Dobin *et al.* 2013) and intron hints generated with the bam2hints tools within the AUGUSTUS program. The gene trained model generated from SNAP and AUSGUSTUS (including the intron–exon boundary hints provided from RNA-seq of *D. stramonium*) was used for a final gene prediction in the program MAKER v.2.31.10 (Campbell *et al.* 2014). Likewise, to help guide the prediction process in MAKER, all the Swiss-Prot peptide sequences from the UniProt database (accessed on 08/06/2021) were also downloaded and used in conjunction with the protein sequences from *A. thaliana*, *C. annuum*, *N. attenuata*, and *S. lycopersicum* (downloaded from EnsemblPlants) to generate peptide evidence in the MAKER pipeline. Only genes that were predicted by both SNAP and AUGUSTUS were retained in the final gene sets. MAKER identifies and masks out repeat elements based on repeat annotation from RepeatMasker and aligns RNA-seq data from the same species and/or related species to the genome (Campbell *et al.* 2014). To help assess the quality of the gene prediction, annotation edit distance (AED) scores were generated for each of the predicted genes as part of the MAKER pipeline (Campbell *et al.* 2014). Genes were further characterized for their putative function by performing a BLAST search (Boratyn *et al.* 2013) of the peptide sequences against the UniProt database. tRNA were predicted using the software tRNAscan-SE v2.05 (Chan *et al.* 2021).

#### DNA extraction, library preparation, for ddRad-sequencing

Genomic DNA (gDNA) was extracted from 163 individuals planted in Teotihuacán and 51 individuals planted in Ticumán. Since we had high mortality of seedlings at the beginning of the experiment in Ticumán, we extracted DNA from more individuals sowed in Teotihuacán (see also De-la-Cruz, Merilä, *et al.* 2020). gDNA was isolated from fresh leaves with a modified CTAB mini-prep protocol for ddRad-seq (Doyle 1991). The total amount of gDNA was measured using Qubit dsDNA HS Assay Kit (Invitrogen, Thermo Fisher Scientific, Waltham, USA). A total of 200 ng of gDNA was used for library preparation. The qualified DNA samples were digested with EcoRI and Hin1II (NlaIII) restriction enzymes (Takara, Osaka, Japan) and subjected to adapter ligation. The digestion and ligation were performed at 37°C for 16 h. The ligation products barcoded with unique P1 adapter were pooled and purified by size selection using E-Gel SizeSelect 2% agarose (Life Technologies, Carlsbad, California, USA). Approximately 400–600 bp fragments were retrieved. The selected size and adaptor-ligated DNA was subsequently amplified by PCR. The PCR products were purified using AMpure XP beads (Beckman Coulter, Brea, California, USA). The purified library was sequenced using Illumina Hiseq X Ten platform (Illumina, San Diego, California, USA). Library preparation and sequencing were carried out by CD Genomics company (Shirley, New York, USA). For the 2 grandparents, gDNA was isolated and measured as above. However, whole genome sequencing was carried out for both, rather than ddRad-seq (De-la-Cruz *et al.* 2021). Libraries were sheared on the Covaris and then prepped for 150PE (paired-end) Illumina HiSeq 4000 sequencing using the KAPA HyperPrep Illumina library prep kits. Final libraries were visualized on the Agilent Fragment Analyzer and then quantified and pooled at equimolar amounts with KAPA qPCR Illumina library quant Universal Kits. The sequencing and library preparations for the grandparents were carried out in the QB3 Functional Genomics and Vincent J. Coates Sequencing Laboratories at the University of California, Berkeley.

#### Variant and genotype calling and linkage map construction

Genotype likelihoods were estimated for each of the individual F_2_ plants as well as both grandparents from Teotihuacán and Ticumán (214 F_2_ plants + grandparents from Teotihuacán and Ticumán). For the genotype likelihood estimations, demultiplexing was initially carried out using Illumina bcl2fastq v2.19 software. This process resulted in the sequence data being returned in fastq format for each individual. Barcodes and indexes had been previously removed by CD Genomics and QB3 services. Subsequently, the Illumina reads underwent trimming, with a Phred quality score threshold of >20 being applied, using TRIMMOMATIC v0.32 (Bolger *et al.* 2014). To ensure data quality, a visual verification step was conducted for the grandparents and ∼80 individual F_2_ plants before and after trimming in FastQC (Andrews 2010). The sequences from each individual plant were then aligned to the chromosome-scale *D. stramonium* reference genome, as described above, utilizing BWA v0.7.17 software with default parameters. The SAM files generated by BWA were converted into BAM format, and these BAM files were sorted using SAMtools v1.10 (Li *et al.* 2009). The calculation of genotype values for each individual was based on the genotype posterior probabilities (GPP). These GPPs were computed using SAMtools mpileup and custom scripts from the Lep-MAP3 program tutorial (Rastas 2017). These scripts were designed to account for alignment quality and involved several filtering steps.

The GPPs for all the F_2_ individuals, as well as a custom pedigree file encompassing the F_2_, F_1_ progeny, and grandparents, were then employed to construct the linkage map in Lep-MAP3. The modules JoinSingles2All and OrderMarkers2 within the Lep-MAP3 pipeline were iterated 5 and 2 times, respectively (Rastas 2017). Subsequently, the “map2genotypes.awk” script was utilized to phase the genotypes. This phasing process ensures that in the output file from the OrderMarkers2 module, one allele is derived from one grandparent, and the other allele originates from the other grandparent (Rastas 2017).

### QTL analysis

The genotypes from 206 individuals, obtained through Lep-MAP3, were employed for the QTL analysis in R/qtl2 v0.28 (Broman *et al.* 2019). First, genotype probabilities conditional on the available marker data, were computed, assuming a genotyping error probability of 0.001. Second, we performed a mixed model (MM) to detect the presence of a QTL. A significant sloped line (*P* = 0.05) in the analysis indicates a difference in the mean phenotype among the 3 genotype groups (AA, AH, BB) for a given marker, signifying the presence of a QTL. In this context, the AA homozygous genotype represented alleles inherited from the grandparent with low resistance (Teotihuacán grandparent), the BB homozygous genotype represented alleles from the grandparent with high resistance (Ticumán grandparent), and AH corresponds to the heterozygote genotype. To account for genetic relatedness between individuals, the MM incorporated a kinship matrix into the analysis. Thus, in the MM, the response variable was plant resistance, while the predictors included genotype, site, and their interaction. The interaction model assessed whether a QTL exhibited different effects in the 2 experimental sites. The statistical significance of the genome scan analysis was assessed through 1,000 permutation tests (Broman *et al.* 2019). Permutation tests determine the maximum logarithm of the odds (LOD) score that could occur by random chance. The genome-wide maximum LOD score was calculated on permuted data, serving as the threshold of statistical significance (*P* = 0.05).

Following the detection of LOD scores from the genome scan and the significance thresholds, we identified QTL peaks associated with plant resistance using the function “find_peaks”. Although high significant LOD scores indicated the vicinity of a QTL, they did not provide its precise position. To pinpoint the exact QTL location, we employed the Bayes credible interval in R/qtl2 (Broman *et al.* 2019). The percentage of the variance explained by the QTL was calculated using the formula:


$$1-10^{\wedge }\;(-(2/n)\;*\;\mathrm{LOD}),$$

where *n* is the sample size and LOD is the LOD score of the significant QTL (see Broman *et al.* 2019). Additive and dominance QTL effects were also calculated for the model. We also retrieved the names of the markers with the highest LOD significant scores to facilitate the identification of candidate genes associated with QTLs. The complete analysis pipeline can be consulted in https://github.com/icruz1989/LinkageMapandQTLanalysis.

### Identification of candidate genes within QTL regions

We retrieved the position of all markers between the Bayes interval obtained from QTL analysis. Then, we looked for the physical positions along the *D. stramonium* genome and retrieved the name of the genes that matched within the interval. Functional annotation of these genes was carried out using the Mercator and MapMan4 pipeline (Schwacke *et al.* 2019).

### Differential expression analysis of herbivory to *L. daturaphila*

We obtained RNA sequences of *D. stramonium* from BioProject DDBJ/ENA/GenBank PRJNA669339. These sequences originated from an experiment involving *D. stramonium* plants exposed to the main folivore, *L. daturaphila* larvae (Coleoptera: Chrysomelidae) (E. Kariñho-Betancourt, N. Calderón Cortés, R. Tapia López, I. De-la-Cruz, J. Núñez-Farfán, K. Oyama, in preparation; De-la-Cruz *et al.* 2022). In this experiment, seeds from 6 plants of the Ticumán population—where the highly resistant parent was selected—were germinated. The seedlings were grown under controlled environmental conditions (16:8 light:dark and 25:20°C light:dark cycle) in the greenhouse of the Institute of Ecology, Universidad Nacional Autónoma de México (E. Kariñho-Betancourt, N. Calderón Cortés, R. Tapia López, I. De-la-Cruz, J. Núñez-Farfán, K. Oyama, in preparation; De-la-Cruz *et al.* 2022). Each seedling was planted in a 150 mL pot filled with sterilized soil and watered as needed. Of these, 3 plants were designated for the damage treatment, exposed to feeding by *L. daturaphila* larvae, and 3 were maintained intact, representing the control treatment (E. Kariñho-Betancourt, N. Calderón Cortés, R. Tapia López, I. De-la-Cruz, J. Núñez-Farfán, K. Oyama, in preparation; De-la-Cruz *et al.* 2022). For the damage treatment, 2 larvae in the second to 4th instar were randomly assigned to the adaxial side of 10 fully expanded leaves. Forty-eight hours later, the larvae were removed, and the leaves they had damaged were collected. Simultaneously, 10 leaves from each control plant were also harvested. These leaves were immediately flash-frozen and stored at −80°C for subsequent RNA extraction and sequencing (E. Kariñho-Betancourt, N. Calderón Cortés, R. Tapia López, I. De-la-Cruz, J. Núñez-Farfán, K. Oyama, in preparation; De-la-Cruz *et al.* 2022).

We then conducted a differential expression analysis comparing the damaged and undamaged plants. To perform this analysis, we aligned the RNA sequences from each treatment (damaged and undamaged plants) to the reference genome using STAR v2.7.10a. We then employed HTSeq v2.0 (Putri *et al.* 2022) to count the number of transcripts mapped to each gene in the improved *D. stramonium* genome. These transcript abundances, obtained through HTSeq, were utilized for the DGEA, which was conducted using DESeq2 v1.34.0 (Love *et al.* 2014). Normalization was carried out using DESeq2’s median of ratios method. We set the significance thresholds at a *P*-value of 0.05 and an adjusted *P*-value of 0.01 (false discovery rate, FDR; Benjamini and Hochberg 1995), along with a log2-fold change (FC) ≥ 1.

To gain insights into the functional roles of the differentially expressed transcripts, we performed enrichment functional annotation using Mercator and MapMan4 (Schwacke *et al.* 2019).

### Gene coexpression network

To identify genes associated with the herbivory response, we utilized the normalized expression profiles. We conducted a pairwise Pearson correlation analysis to construct a network, retaining only significant correlations (*P* = 0.05) while controlling for the Benjamini–Hochberg FDR (Benjamini and Hochberg 1995). Network statistics, including connectedness, betweenness centrality, and average rank for both statistics, were computed using the R-igraph v1.3.5 software (Csardi and Nepusz 2005). For visualization and clustering (utilizing community clustering with GLay), we employed Cytoscape v3.9.1 (Shannon *et al.* 2003).

### Protein–protein interaction network

We constructed protein–protein interaction networks using genes identified within the QTL region and those that were overexpressed. This analysis was conducted with default parameters on the STRING-db platform (Szklarczyk *et al.* 2019). These networks enabled us to uncover functional associations among proteins involved in the resistance mechanisms against herbivores in *D. stramonium*. We performed enrichment analysis within the same program, utilizing gene ontology and Kyoto Encyclopedia of Genes and Genomes (KEGG) (Szklarczyk *et al.* 2019). We set the network association algorithm with a significance threshold of *P* = 0.001.

## Results

### Scale-chromosome genome assembly

PacBio Sequel I and RNA sequences were used to improve the scaffolding of the genome before the final genome assembly by Dovetail Genomics (see below). This improvement resulted in a genome of 23,934 scaffolds (Table 1). Subsequently, this draft genome was used as anchor for the HiRise Dovetail assembly. For this final assembly, we used 127,475,161 Omni-C paired-end sequences, obtaining a scale-chromosome genome assembly of 6,109 scaffolds (Table 1; Fig. 1b). HiRise scaffolding joined 18,023 contigs. The total length of the assembly was 1.59 Gb (Table 1; Fig. 1b and c). The assemblies showed a normal pattern for relative GC content. The total number of scaffolds > 1,000 bp was 6,045 (Fig. 1c). The largest scaffold of the assembly had a size of 172 Mb. N50 and N90 scaffold lengths resulted in 124,029,016 (124 Mb) and 68,742,999 bp (68 Mb), respectively (Table 1). In contrast, L50 and L90 metrics were 6 and 12 scaffolds, respectively (Fig. 1b). The *Datura* genome assembly covered 97.25% complete single-copy orthologs (BUSCOs) of the 255 total BUSCOs searched (Table 1; Fig. 1d). Complete duplicated and fragmented BUSCOS covered 14.90 and 0.15%, respectively (Fig. 1d). Only 4 single-copy orthologs were found as fragmented by the BUSCO program (Table 1; Fig. 1d). We also mapped the raw PE Omni-C sequences to the new genome assembly for genome validation quality; the overall mapping rate was 99.83%.

**Table 1.** Assembly statistics of the previously published and a new 1 genome of *D. stramonium*, annotation statistics for the *Datura stramonium* genomes, and BUSCO statistics for both genomes. ^a^The published draft genomes can be found in De-la-Cruz *et al.* (2021)

	Published genome*^a^*	New genome
Assembly		
Total length	1,588,447,316	1,590,250,582
N50	110,881	124,029,016
L50	3,749	6
N90	31,242	68,742,999
L90	14,149	12
Largest scaffold	3,149,502	172,102,712
Number of scaffolds	23,934	6,109
Number of scaffolds > 1 kbp	23,870	6,045
Number of gaps	7,268	25,291
Number of N’s per 100 kbp	1,805.11	1,916.46
Annotation		
% of the repeats	76.04	78.65%
Total number of genes	33,856	40,229
BUSCO statistics		
Complete BUSCOs (C)	246 (96.47%)	248 (97.25%)
Complete and single-copy BUSCOs (S)	211 (82.75%)	210 (82.35%)
Complete and duplicated BUSCOs (D)	35	38
Fragmented BUSCOs (F)	5	4

The annotation pipeline included 40,229 protein-coding genes (Table 1). The total coding region included 53,417,445 bp (Table 1). Average length of genes consisted of 1,327 bp. The total number of single exons was 2,167 (Table 1). A total of 99% gene models showed high confidence matches (E-value ≤ 1e^−5^) in the UniProtKB/TrEMBL database. Repetitive elements covered the 78.65% of the genome (Table 1).

### Genetic basis of plant resistance

**Fig. 2.**
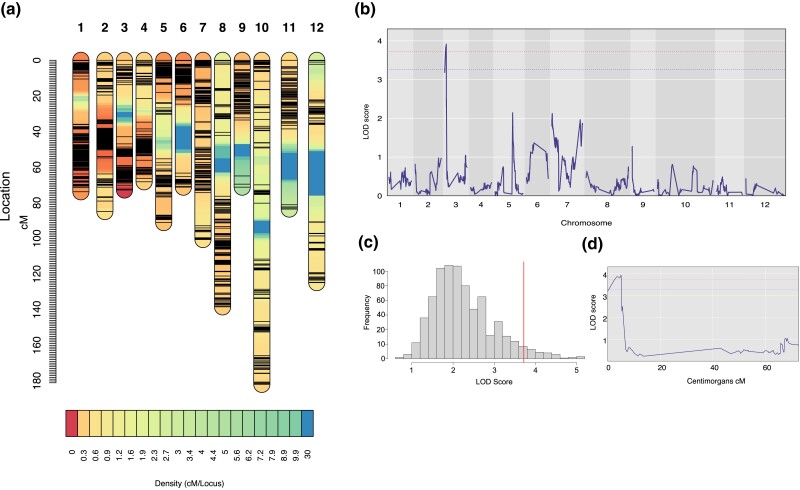
Density linkage map of *D. stramonium* (a) showing the density and position of the markers (SNPs) across the 12 chromosomes (LGs). The first chromosome contains the highest number of markers (*n* = 6,006). b) QTL analysis for plant resistance of *D. stramonium*, revealing a single significant QTL at chromosome 3 (LOD score = 3.9). c) Permutation test (1,000 iterations) for the QTL analysis, revealing a cutoff LOD score of 3.9 for plant resistance. d) Close-up for the highest LOD score for plant resistance at chromosome 3, position 3.9 cM. Red and blue lines depict the *P*-value threshold (0.05 and 0.07, respectively). The Bayes interval of the QTL region ranges from 0 to 5.176 cM.

The construction of a linkage map using the new genome resulted in the clustering of nearly all single nucleotide polymorphism markers (*n* = 20,053 SNPs) into 12 linkage groups (LGs), hereinafter referred to as chromosomes (Fig. 2a). The first chromosome (Chr1) exhibited the highest marker count (*n* = 6,006 markers), with the count gradually decreasing to (Chr12), which contained 141 markers (Fig. 2a; Supplementary Table 1). The average distance between markers was 0.25 cM, contributing to a cumulative map length of 1,162.4 cM (Fig. 2a, Supplementary Table 1).

**Fig. 3.**
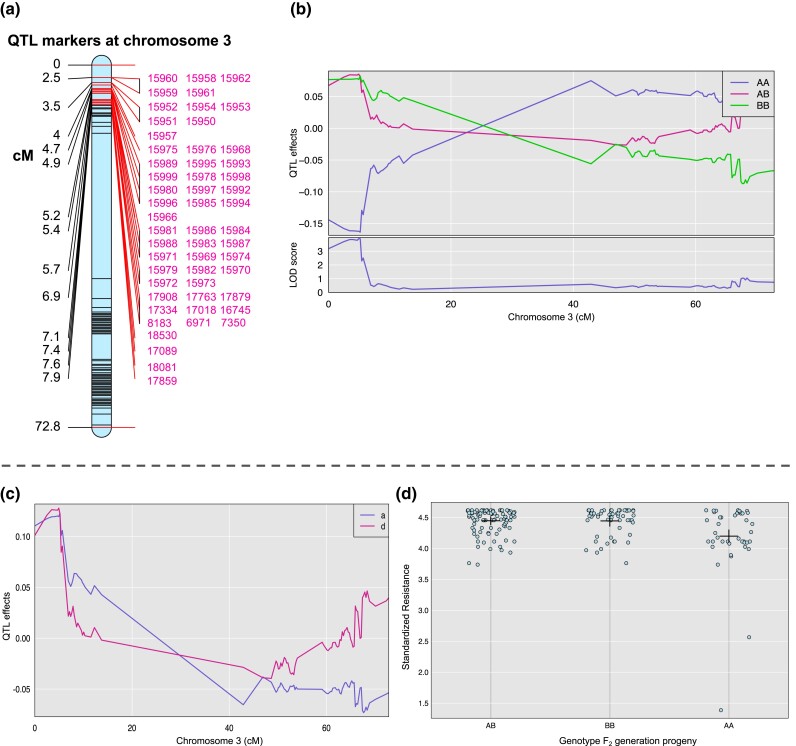
a) Distribution of markers across the QTL region (0–5.176 cM) for plant resistance against chewing herbivores at chromosome 3. b) QTL effects for plant resistance of *D. stramonium* at chromosome 3 plotted by genotypes. A and B alleles were inherited from the Teotihuacán (low resistance) and Ticumán (high resistance) grandparents to produce the F_2_ progeny, respectively. c) Additive (a) and dominance (d) effects of the QTL, revealing a dominance effect of the allele B, which was inherited from the Ticumán parent. d) Relationship between the genotypes and plant resistance (untransformed data); mean and standard errors are also shown in the figure. Genotype AA has lower resistance than BB and AB genotypes.

We identified a single QTL associated with plant resistance in *D. stramonium*, located on chromosome 3 (Fig. 2b). The explained variance by the QTL was 8.44%. The highest LOD score was detected at position 4.93 cM with a value of 3.91 (Fig. 2c and d). The Bayesian credible interval for the QTL region spanned from 0 to 5.17 cM (Figs. 2d and 3a). We retrieved all markers and genes within the QTL region, finding 54 markers and 127 genes within this specific interval (Table 2, Supplementary Table 2, Fig. 3a). The QTL effects of plant resistance were more pronounced in genotypes BB (where the B allele was inherited from the Ticumán grandparent, chosen for its high resistance) and AB (where the A allele was inherited from the Teotihuacán grandparent, chosen for its low resistance), as compared with the AA genotype (Fig. 3b). This suggests that the BB and AB genotypes demonstrated enhanced resistance relative to the AA genotype (Fig. 3b). A dominance effect was identified for the B allele (Fig. 3b to d). The gene GDSL sterase/lipase LIP-4 was the closest gene to the highest QTL peak (marker 15,992 at position 4.93 cM; Table 2, Supplementary Table 2). We did not observe an interaction between the site and significant markers within the highest LOD peak of the QTL region.

**Table 2.** Significant markers (SNPs) associated with the QTL of plant resistance at chromosome 3 (position = 4.93 cM) in *Datura stramonium* within a region of 2 Mb from the highest LOD score (3.91). Marker “15,992” was the closest one to the highest LOD score

Marker	*P*-value	LogWorth	Effect size	Y mean	SSE	DFE	MSE	*F* ratio	*R* square
15,966	9.23E^−05^	4.03	0.40	4.39	20.36	196	0.10	9.74	0.09
15,978	9.32E^−05^	4.03	0.40	4.39	20.36	196	0.10	9.73	0.09
15,980	9.32E^−05^	4.03	0.40	4.39	20.36	196	0.10	9.73	0.09
15,985	9.32E^−05^	4.03	0.40	4.39	20.36	196	0.10	9.73	0.09
15,989	9.32E^−05^	4.03	0.40	4.39	20.36	196	0.10	9.73	0.09
15,992	9.32E^−05^	4.03	0.40	4.39	20.36	196	0.10	9.73	0.09
15,993	9.32E^−05^	4.03	0.40	4.39	20.36	196	0.10	9.73	0.09
15,994	9.32E^−05^	4.03	0.40	4.39	20.36	196	0.10	9.73	0.09
15,995	9.32E^−05^	4.03	0.40	4.39	20.36	196	0.10	9.73	0.09
15,996	9.32E^−05^	4.03	0.40	4.39	20.36	196	0.10	9.73	0.09
15,997	9.32E^−05^	4.03	0.40	4.39	20.36	196	0.10	9.73	0.09
15,998	9.32E^−05^	4.03	0.40	4.39	20.36	196	0.10	9.73	0.09
15,999	9.32E^−05^	4.03	0.40	4.39	20.36	196	0.10	9.73	0.09
15,950	0.000111	3.95	0.40	4.39	20.40	196	0.10	9.54	0.08
15,951	0.000111	3.95	0.40	4.39	20.40	196	0.10	9.54	0.08
15,952	0.000111	3.95	0.40	4.39	20.40	196	0.10	9.54	0.08
15,953	0.000111	3.95	0.40	4.39	20.40	196	0.10	9.54	0.08
15,954	0.000111	3.95	0.40	4.39	20.40	196	0.10	9.54	0.08
15,957	0.000111	3.95	0.40	4.39	20.40	196	0.10	9.54	0.08
15,968	0.000132	3.87	0.39	4.39	20.44	196	0.10	9.35	0.08
15,975	0.000132	3.87	0.39	4.39	20.44	196	0.10	9.35	0.08
15,976	0.000132	3.87	0.39	4.39	20.44	196	0.10	9.35	0.08
15,958	0.00015	3.82	0.39	4.39	20.46	196	0.10	9.21	0.08
15,959	0.00015	3.82	0.39	4.39	20.46	196	0.10	9.21	0.08
15,960	0.00015	3.82	0.39	4.39	20.46	196	0.10	9.21	0.08
15,961	0.00015	3.82	0.39	4.39	20.46	196	0.10	9.21	0.08
15,962	0.00015	3.82	0.39	4.39	20.46	196	0.10	9.21	0.08
15,949	0.000387	3.41	0.37	4.39	20.66	196	0.10	8.18	0.07
15,970	0.002188	2.65	0.33	4.39	21.03	196	0.10	6.32	0.06
15,972	0.002188	2.65	0.33	4.39	21.03	196	0.10	6.32	0.06
15,973	0.002188	2.65	0.33	4.39	21.03	196	0.10	6.32	0.06
15,979	0.002188	2.65	0.33	4.39	21.03	196	0.10	6.32	0.06
15,982	0.002188	2.65	0.33	4.39	21.03	196	0.10	6.32	0.06
15,969	0.00332	2.47	0.31	4.39	21.12	196	0.10	5.87	0.05
15,971	0.00332	2.47	0.31	4.39	21.12	196	0.10	5.87	0.05
15,974	0.00332	2.47	0.31	4.39	21.12	196	0.10	5.87	0.05
15,981	0.00332	2.47	0.31	4.39	21.12	196	0.10	5.87	0.05
15,983	0.00332	2.47	0.31	4.39	21.12	196	0.10	5.87	0.05
15,984	0.00332	2.47	0.31	4.39	21.12	196	0.10	5.87	0.05
15,986	0.00332	2.47	0.31	4.39	21.12	196	0.10	5.87	0.05
15,987	0.00332	2.47	0.31	4.39	21.12	196	0.10	5.87	0.05
15,988	0.00332	2.47	0.31	4.39	21.12	196	0.10	5.87	0.05
7277	0.004919	2.30	0.30	4.39	21.20	196	0.10	5.46	0.05
7302	0.009226	2.03	0.29	4.39	21.34	196	0.10	4.79	0.04

Enrichment functional annotation using Mercartor and MapMan4 for the genes found within the QTL region unveiled that the majority of these genes was associated with various biological functions, including enzymatic activity, nutrient uptake, solute transport, cell wall organization, protein modification, DNA damage response, and coenzyme metabolism (Supplementary Table 2 and Fig. 1).

**Fig. 4.**
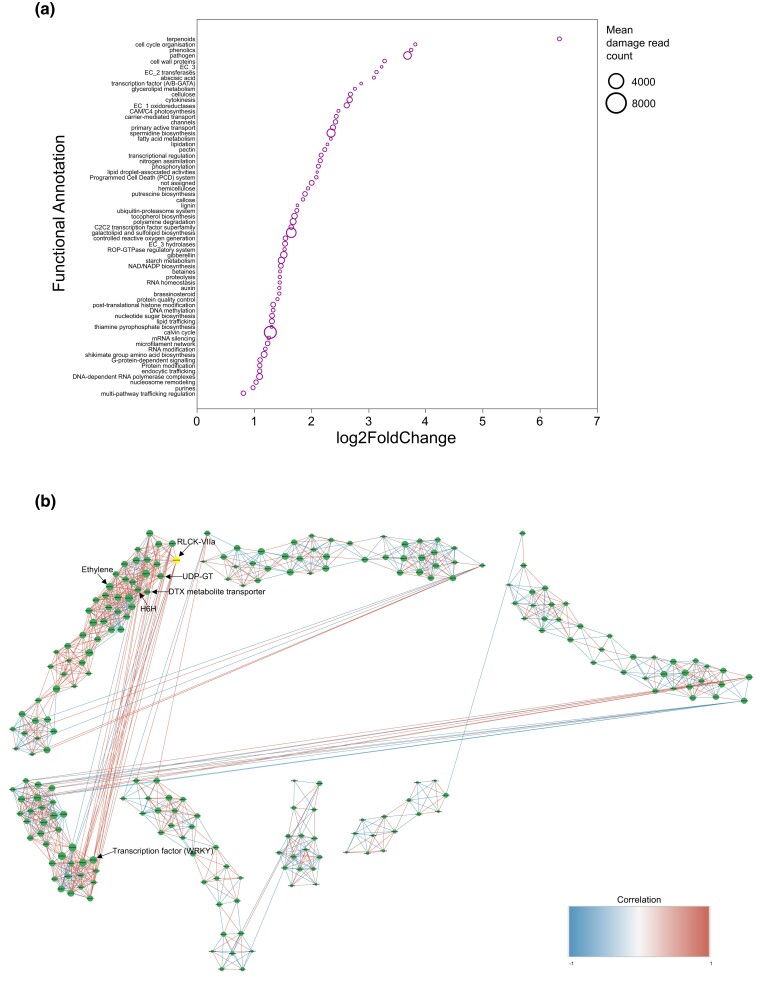
a) Dot plot illustrating the Log2FoldChange of enriched overexpressed genes from the DGEA between undamaged and damaged plants by the larvae of *L. daturaphila* (the most dangerous folivore of *D. stramonium*). Natural plants from the Ticumán population were used for the DGEA. Functional annotation was performed by MapMan4. The size of the circle represents the mean transcript counts for plant damage. The plot is ordered in ascending mode from the highest Log2FoldChange. b) Gene coexpression network analysis showing the relationships between the coexpressed genes. In yellow color is highlighted the receptor-like protein kinase (RLCK-VLLa) gene that was either found within the QTL region (0–5.176 cM) or overexpressed in the DGEA between undamaged and damaged plants from the Ticumán population of *D. stramonium*. This gene is highly connected to important genes that have been related to plant resistance in *D. stramonium*, such as UDP-glycosyltranferase, ethylene response and H6H, DTX metabolite transporter, and the transcription factor WRKY. The gene RLCK-VLLa connects 2 modules of genes in the network via the transcription factor WRKY. Red and blue lines show positive and negative correlations, respectively. See also Fig. 5 and Supplementary Fig. 2.

Differential expression analysis identified a total of 221 genes displaying signals of overexpression, indicating significant differential expression between control and damage conditions (Supplementary Table 3). Annotation using MapMan4 of these overexpressed genes revealed that the majority of these genes was associated with various biological processes, including enzymatic activity, polyamine metabolism, secondary metabolism, solute transport, carbohydrate and lipid metabolism, as well as cell wall organization (Supplementary Table 3 and Fig. 1). Among the highly overexpressed genes, there was a notable presence of those involved in the biosynthesis of phenylalanine, terpenoids, tropane alkaloids, carotenoids, and the pentose pathway (Supplementary Table 3). The genes with the most significant Lod2fold change included terpenoid phytoene synthase (PSY), the pathogen-related gene defensin (PDF2), and the receptor-like protein kinase (RLCK-VIIa) (Fig. 4a, Supplementary Table 3). We also found multiple receptor-like protein kinases coexpressed with central genes for the tropane alkaloid biosynthesis [e.g. hyoscyamine 6-dioxygenase-like (H6H)] and for the terpenoid biosynthesis, like the UDP-glycosyltransferase (Fig. 4b).

**Fig. 5.**
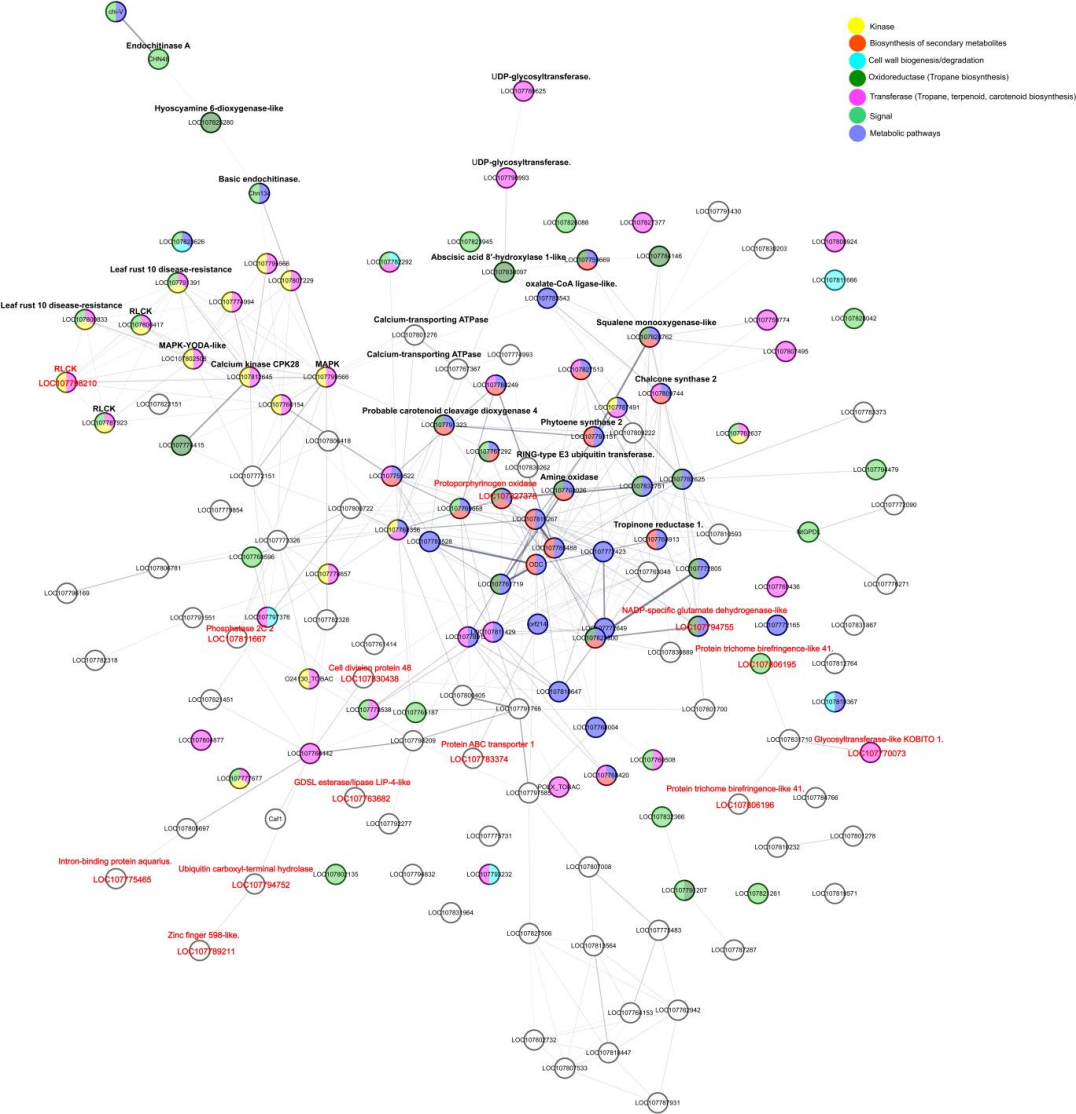
Protein–protein interaction subnetwork constructed in STRING-db using both the genes detected within the QTL region and overexpressed genes from the differential expression analysis between damage and undamaged plants by the larvae of *L. daturaphila*. According with gene ontology, this subnetwork was classified within catalytic activity. Important genes related to the plant resistance machinery were detected within this subnetwork. Genes related to signaling of plant damage and involved in the biosynthesis of tropane alkaloids, terpenoids, and carotenoid biosynthesis were associated in this network. See also Supplementary Fig. 2.

To clarify potential interactions among genes implicated in plant defense mechanisms, we constructed a protein–protein interaction network. This network was built utilizing genes from the QTL and, guided by the results of the differential gene expression analyses, delineated 4 distinct functional enrichments. These were categorized based on gene ontology into the following functional groups: NA-directed DNA polymerase activity, transferase activity, catalytic activity, and cellular anatomical entity (Supplementary Fig. 2). Within the subnetwork related to catalytic activity, we identified 13 genes that were not only present in the QTL analysis but were also associated with the biosynthesis of tropane alkaloids, terpenoids, and carotenoids pathways (Fig. 5). We identified 5 genes that were shared between the QTL region on chromosome 3 (interval 0–5.17 cM) and the differential expression analysis (Supplementary Tables 2 and 3). These genes include the borate transporter (BOR), C2H2 zinc finger transcription factor, calcium-permeable channel, receptor-like protein kinase (RLCK-VIIa), and transcription factor bZIP (Supplementary Tables 2 and 3).

## Discussion

### Improving the genome assembly

We successfully improved the Mexican genome of *D. stramonium* to chromosome-scale resolution. The earlier published *D. stramonium* genome consisted of 27,215 contigs, with an N50 metric of 84,121 bp (De-la-Cruz *et al.* 2021). Through the implementation of our workflow, incorporating Omni-C and HiRise scaffolding sequences, we were able to join 18,023 contigs. L90 statistics resulted in 12 scaffolds, each corresponding to the known chromosomes of *D. stramonium* (Satina *et al.* 1941; Zhang *et al.* 2023).

Assessing the quality of our assembly, we found that the *Datura* genome encompassed 97.25% of complete single-copy orthologs (BUSCOs) out of the 255 total BUSCOs searched. Only 5 single-copy orthologs exhibited fragmentation, a level of completeness consistent with both the initial Mexican draft genome and the newly published Chinese *D. stramonium* genome (De-la-Cruz *et al.* 2021; Zhang *et al.* 2023). This consistency in quality assembly statistics emphasizes the robustness and completeness of our assembly. Furthermore, we constructed a dense linkage map that aligns with the expected haploid number of chromosomes in *Datura* (Satina *et al.* 1941; Zhang *et al.* 2023).

### Genetic basis of plant resistance

The protein–protein interaction network that we constructed using genes from the QTL region, along with the overexpressed genes, unveiled that 13 of the genes located within the QTL region were linked to a subnetwork categorized under catalytic activity based on gene ontology classification (Ashburner *et al*. 2000; Fig. 5). This subnetwork is intricately associated with the biosynthesis of phenylalanine, terpenoids, carotenoids, and tropane alkaloids (Fig. 5). Within this subnetwork, it appears that receptor-like protein kinases (RLCKs) play a pivotal role in controlling the signaling transduction pathways associated with the biosynthesis of these secondary compounds. Receptor-like protein kinases have previously been linked to herbivory and pathogen infections (Morris and Walker 2003; Afzal *et al.* 2008; Jurca *et al.* 2008; Kourelis and van der Hoorn 2018). In fact, numerous studies have emphasized the role of plant receptor-like kinases as crucial “sentinels” in orchestrating plant defense responses, as we observed in our study (Goff and Ramonell 2007; Kourelis and van der Hoorn 2018). For example, our investigation identified several RLCKs, including 1 gene located within the QTL region (i.e. RLCK-VIIa), that were directly associated with the LEAF RUST 10 DISEASE-RESISTANCE LOCUS (UniProt ID LOC107791391, LOC107800833). Leaf rust, caused by a group of related fungi in the order *Uredinales* or *Pucciniales*, is a common fungal disease affecting various plant species, including those from the Solanaceae family (Helfer 2014).

The LEAF RUST LOCUS was also found to be associated with and coexpressed alongside mitogen-activated protein kinases, calcium-dependent protein kinases, and endochitinases. All these genes have been categorized in gene ontology as components of the plant defense response, particularly against pathogens (Ashburner *et al*. 2000). Furthermore, the gene H6H exhibited an association with RLCKs, and the strongest connection between H6H and RLCKs was mediated through the mitogen-activated protein kinase (LOC107799566) and endochitinase genes. H6H was also highly coexpressed with the gene RLCK-VII, which was found within the QTL region. H6H catalyzes the production of scopolamine in a few Solanaceous plants, such as *Atropa belladonna*, *D. stramonium*, *Hyoscyamus niger*, and *Mandragora officinarum* (Hashimoto *et al.* 1991; Lounasmaa and Tamminen 1993; O’Hagan 2000; Humphrey and O’Hagan 2001; Jirschitzka *et al.* 2012; Zhang *et al.* 2023). In particular, scopolamine is a tropane alkaloid associated with resistance to herbivores in *D. stramonium* (Castillo *et al.* 2013; [Bibr jkad288-B12][Bibr jkad288-B69]; De-la-Cruz, Merilä, *et al.* 2020). In a previous study with these same F_2_ progeny studied here, we observed that plants with higher scopolamine concentration had lower number of adult *Trichobaris soror* insects on leaves (De-la-Cruz, Merilä, *et al.* 2020). A previous investigation has demonstrated that plants with higher concentrations of scopolamine in their seeds exhibited a reduction in the number of *T. soror* larvae in *D. stramonium*. Moreover, these plants preserved a higher number of seeds subsequent to the larval predation (Miranda-Pérez *et al.* 2016).

We also observed that the overexpressed gene mitogen-activated protein kinase (LOC107799566), which was associated with all the RLCK genes, also was associated with the enzyme amine oxidase (LOC107768026). Amine oxidase enzyme plays a pivotal role in the biosynthesis of tropane alkaloids, leading to the production of 1-methylpyrrolinium, a simple pyrrolidine alkaloid and precursor of 4-(1-methyl-2-pyrrolidinyl)-3-oxobutanoate (Kanehisa and Goto 2000). This compound serves as the substrate for the synthesis of tropinone via the enzymatic activity of chalcone synthase, which was also connected with amine oxidase in our study (Kanehisa and Goto 2000). Moreover, chalcone synthase was also associated with tropinone reductase I, an enzyme responsible for tropine production (Kanehisa and Goto 2000; Dräger 2006; Parks *et al.* 2023). Tropine serves as an intermediate substrate in the synthesis of atropine and scopolamine (Richter *et al.* 2005). Remarkably, the association between the mitogen-activated protein kinase, RLCK genes, and amine oxidase occurred through 2 possible ways: one involving calcium-transporting ATPase (LOC107801276) and the other involving chalcone synthase 2 (LOC107809744), RING-type E3 ubiquitin transferase (LOC107830262), and oxalate-CoA ligase-like (LOC107783543). These latter 3 genes have been implicated as messengers in response to the perception of pathogen-associated molecular patterns (PAMPs) (Lecourieux *et al.* 2006; Cheval *et al.* 2013; Hagihara *et al.* 2022). Thus, the collective presence of these genes involved in the biosynthesis of tropane alkaloids in our protein–protein interaction network strongly suggests that tropane alkaloids have evolved as defense mechanisms against the specialist herbivores in *D. stramonium*. Nevertheless, we do not discard that these defenses may also be effective against pathogens and viruses.

Within the same catalytic activity subnetwork, our findings revealed connections between terpenoid genes and RLCKs, primarily mediated by the same mitogen-activated protein kinase (LOC107799566) that linked tropane alkaloid genes. Notably, this kinase exhibited associations with sesquiterpene genes, particularly abscisic acid 8′-hydroxylase 1-like (LOC107830097). The latter gene, in turn, was connected with UDP-glycosyltransferases (UDP-GT; LOC107790993, LOC107789625) and squalene monooxygenase-like (LOC107828762). These latter 2 genes play pivotal roles in glycosylation and terpenoid biosynthesis (Langlois-Meurinne *et al.* 2005; Dong *et al.* 2018). It has been demonstrated that the coexpression of squalene monooxygenases leads to increased triterpenoid production in plants (Dong *et al.* 2018). In previous investigations, we have observed that UDP-glycosyltransferases and several terpene gene families are overrepresented and exhibit signals of positive selection and expansion in the *D. stramonium* genome across the Solanaceae family (De-la-Cruz *et al.* 2021, 2022). Furthermore, we have demonstrated that the highest concentration of a major triterpenoid produced by the same F_2_ progeny used in this study led to a reduction in the population of the most harmful herbivore of *D. stramonium*, the larvae of *L. daturaphila* (De-la-Cruz, Merilä, *et al.* 2020). These F_2_ plants also exhibited reduced leaf area consumption by chewing herbivores and were genetically closer to the Ticumán parent (i.e. F_2_ plants with the BB genotype), which was selected for its high resistance (De-la-Cruz, Cruz, *et al.* 2020, De-la-Cruz, Merilä, *et al.* 2020). Additionally, phenotypic selection analyses unveiled that this compound increased seed production and the survival rate of these highly resistant F_2_ plants (De-la-Cruz, Merilä, *et al.* 2020).

We then hypothesize that RLCKs play a key role in signaling and regulating tropane alkaloids and terpenoids. Among these, 2 highly interconnected RLCK genes, RLCK-VII and mitogen-activated protein kinase, emerge as central components in the signaling and regulation of defense mechanisms in *D. stramonium*. We speculate that these genes control the activation of specific defense responses against the most destructive herbivores of *D. stramonium*. For instance, they may induce scopolamine production as a defense against the larvae of the seed predator *T. soror* and promote the synthesis of terpenoids to deter the most damaging folivore, the larvae of *L. daturaphila*.

We also identified the TRICHOME BIREFRINGENCE-LIKE gene within the QTL region. TRICHOME BIREFRINGENCE is responsible for trichome production in plants, which are often considered as the first line of defense against insect herbivores and pathogens (Levin 1973; Mauricio 2005; Bischoff *et al.* 2010; Tian *et al.* 2012; Kaur and Kariyat 2023). Trichomes can impede the movement of insect herbivores, reducing leaf consumption, and even causing damage to herbivore gut linings (Weinhold and Baldwin 2011; Kariyat *et al.* 2017; Kaur and Kariyat 2023). In *D. stramonium*, previous research has shown that trichomes contribute to resistance against folivores like *L. daturaphila* and *Epitrix parvula*. It has been also reported that plants with a higher number of trichomes had higher fitness in the Ticumán population, the locality of the highly resistant grandparent used to produce the F_2_ progeny (Valverde *et al.* 2001; Kariñho-Betancourt and Núñez-Farfán 2015).

Our results also revealed that the GDSL esterase/lipase LIP-4 (LOC107763682) was the nearest gene to the highest peak within the QTL region. This gene was only directly connected in the primary protein–protein interaction network with the BES1/BZR1 homolog protein 4-like isoform X1 (LOC107790592). The latter gene was further associated with the zinc finger protein ZAT10-like (LOC107810271) and the dirigent protein (LOC107796045). These genes have been linked to abiotic stress responses such as drought and salt stress (Han *et al.* 2020), but, to the best of our knowledge, it has not been associated with herbivory. Furthermore, in *A. thaliana* and *Capsicum annum* L (Solanaceae), it has been demonstrated that 2 GDSL LIPASEs, GLIP1 and GLIP2, play crucial roles in resistance to pathogens, including *Alternaria brassicicola* and *Erwinia carotovora* (Oh *et al.* 2005; Hong *et al.* 2008; Kim *et al.* 2008; Lee *et al.* 2009).

In summary, our results suggest that the resistance mechanism of *D. stramonium* is influenced by the complex interplay of multiple genes with minor effects. The dominance effect detected in the QTL implies that natural selection favors allelic variants that enhance resistance in this species, ultimately contributing to its fitness. This adaptation may lead to the development of novel defense strategies against the specialized herbivores that affect *D. stramonium*. The identification of QTLs and candidate resistance genes in this species has the potential to provide valuable tools for accelerating the development of crop varieties within the Solanaceae plant family, which holds economic significance. Furthermore, the genomic and phenotypic data generated by this study will serve as fundamental resources for investigating the evolution and ecology of important secondary compounds, such as tropanes and terpenoids, as well as the genes responsible for mediating plant–insect interactions.

## Supplementary Material

jkad288_Supplementary_Data

## Data Availability

Scripts, commands, and the entire workflow used for the bioinformatic analyses presented in this manuscript can be consulted at https://github.com/icruz1989/. ddRad-seq data has been deposited at DDBJ/ENA/GenBank under the BioProject PRJNA663170. The full genome sequence and its annotation has been deposited at GenBank (DDBJ/ENA/GenBank BioProject PRJNA907288). In addition, we have deposited all supplementary data supporting this research at the GSA figshare portal. The deposited data include the genome assembly and annotation, phenotypic and genotypic data, linkage map, proteins in a fasta file, chromosome lengths of the genomes, marker positions and sequences in a fasta file specifically within the QTL region, and RNA counts for DGEA. Access to these data can be found at figshare: https://doi.org/10.25387/g3.23283128. Supplemental material available at G3 online.
