# Supplementary material for: The chromosome-scale genome and the genetic resistance machinery against insect herbivores of the Mexican toloache, Datura stramonium
Source: G3 (Bethesda). 2023 Dec 19;14(2):jkad288. doi: 10.1093/g3journal/jkad288 (PMC10849327; doi:10.1093/g3journal/jkad288)

Figure S5. Protein-protein interaction network constructed in STRING-db using both the genes detected withing the QTL region and overexpressed genes from the differential expression analysis between damage and undamaged plants by the larvae of *Lema daturaphila*. According with gene ontology, this network contains four different functional enrichments classified within RNA-directed DNA polymerase activity, transferase activity, cellular anatomical entity and catalytic activity. Important genes related to the plant resistance machinery were detected within this network. The gene GDSL sterase/lipase LIP-4 was the closest gene to the highest QTL peak (marker 15,992 at position 4.93 cM). See also Figure 5. The interactive link to consult the full network can be found in https://version-12-0.string-db.org/cgi/network?networkId=bmMaUyVvt74M full.

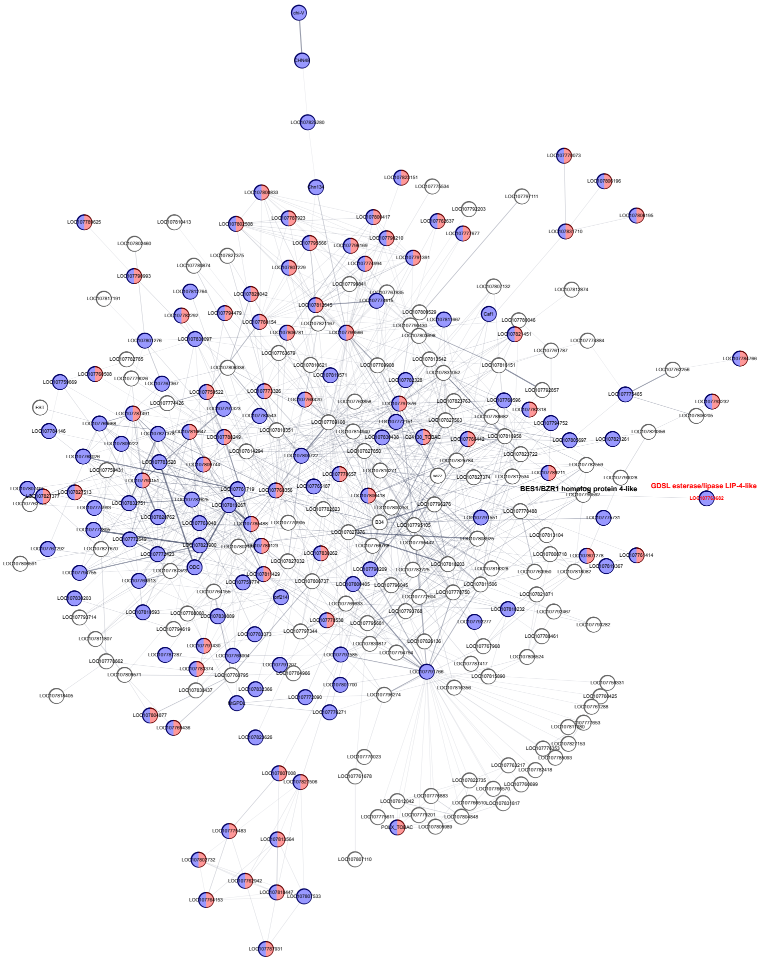

Supplement: jkad288_Supplementary_Data [file jkad288_supplementary_data.zip › Figure_S2_G3-2023-404717.pdf]
