# Supplementary material for: The chromosome-scale genome and the genetic resistance machinery against insect herbivores of the Mexican toloache, Datura stramonium
Source: G3 (Bethesda). 2023 Dec 19;14(2):jkad288. doi: 10.1093/g3journal/jkad288 (PMC10849327; doi:10.1093/g3journal/jkad288)
Supplement: jkad288_Supplementary_Data [file jkad288_supplementary_data.zip › Table_S2_G3-2023-404717.pdf]

Table S2. Functional Mapman4 annotation of genes within the QTL region 0-5.176 cM according to the Bayes Interval.

BIN Number	Full Hierarchical Annotation of Mapmap4	Gene Name	Description of Gene Functionality
'13.3.5.2.1.1.1'	'Cell division.meiotic recombination.meiotic crossover.class II interference-insensitive crossover pathway.non-canonical Holliday junction resolution.MUS81-EME1 Holliday junction cleavage heterodimer.component *(MUS81)'	'ann06028'	'mercator4v5.0: component *(MUS81) of MUS81-EME1 Holliday junction cleavage heterodimer & swissprot: Crossover junction endonuclease MUS81 & original description: none'
'13.3.5.2.1.1.1'	'Cell division.meiotic recombination.meiotic crossover.class II interference-insensitive crossover pathway.non-canonical Holliday junction resolution.MUS81-EME1 Holliday junction cleavage heterodimer.component *(MUS81)'	'ann06025'	'mercator4v5.0: component *(MUS81) of MUS81-EME1 Holliday junction cleavage heterodimer & swissprot: Crossover junction endonuclease MUS81 & original description: none'
'13.3.1.4'	'Cell division.meiotic recombination.meiotic initiation.chromosome axis protein *(ASY3)'	'ann06076'	'mercator4v5.0: meiotic recombination chromosome axis protein *(ASY3) & swissprot: Meiosis-specific protein ASY3 & original description: none'
'21.1.2.3'	'Cell wall organisation.cellulose.cellulose-hemicellulose network assembly.regulatory protein *(KOBITO)'	'ann06001'	'mercator4v5.0: regulatory protein *(KOBITO) of cellulose-hemicellulose network assembly & swissprot: Glycosyltransferase-like KOBITO 1 & original description: none'
'21.3.5.1.4'	'Cell wall organisation.pectin.modification and degradation.polygalacturonase activities.polygalacturonase *(PGX3-like)'	'ann06110'	'mercator4v5.0: polygalacturonase *(PGX3-like) & swissprot: Polygalacturonase At1g48100 & original description: none'
'12.1.1.3'	'Chromatin organisation.chromatin structure.DNA wrapping.histone *(H3)'	'ann06080'	'mercator4v5.0: histone *(H3) & swissprot: Histone H3.2 & original description: none'
'12.1.1.3'	'Chromatin organisation.chromatin structure.DNA wrapping.histone *(H3)'	'ann06079'	'mercator4v5.0: histone *(H3) & swissprot: Histone H3.2 & original description: none'
'12.4.8.2.3'	'Chromatin organisation.nucleosome remodeling.other chromatin remodeling activities.SSO1653-like group.chromatin remodeling factor *(ERCC6)'	'ann06016'	'mercator4v5.0: chromatin remodeling factor *(ERCC6) & swissprot: Protein CHROMATIN REMODELING 24 & original description: none'
'7.8.3.6.2'	'Coenzyme metabolism.prenylquinone biosynthesis.ubiquinone biosynthesis.accessory protein activities.ABC1-type kinase *(COQ8)'	'ann06074'	'mercator4v5.0: ABC1-type kinase *(COQ8) & swissprot: Protein ABC transporter 1, mitochondrial & original description: none'
'7.13.4.3'	'Coenzyme metabolism.tetrapyrrole biosynthesis.protoporphyrin IX formation.protoporphyrinogen IX oxidase'	'ann06077'	'mercator4v5.0: protoporphyrinogen IX oxidase & swissprot: Protoporphyrinogen oxidase, chloroplastic & original description: none'
'50.1.4'	'Enzyme classification.EC_1 oxidoreductases.EC_1.4 oxidoreductase acting on CH-NH2 group of donor'	'ann06009'	'mercator4v5.0: EC_1.4 oxidoreductase acting on CH-NH2 group of donor & swissprot: NADP-specific glutamate dehydrogenase (Fragment) & original description: none'
'50.1.4'	'Enzyme classification.EC_1 oxidoreductases.EC_1.4 oxidoreductase acting on CH-NH2 group of donor'	'ann06006'	'mercator4v5.0: EC_1.4 oxidoreductase acting on CH-NH2 group of donor & swissprot: NADP-specific glutamate dehydrogenase (Fragment) & original description: none'
'50.2.1'	'Enzyme classification.EC_2 transferases.EC_2.1 transferase transferring one-carbon group'	'ann06058'	'mercator4v5.0: EC_2.1 transferase transferring one-carbon group & swissprot: Trans-resveratrol di-O-methyltransferase & original description: none'
'50.2.1'	'Enzyme classification.EC_2 transferases.EC_2.1 transferase transferring one-carbon group'	'ann06045'	'mercator4v5.0: EC_2.1 transferase transferring one-carbon group & swissprot: Trans-resveratrol di-O-methyltransferase & original description: none'
'50.2.7'	'Enzyme classification.EC_2 transferases.EC_2.7 transferase transferring phosphorus-containing group'	'ann06015'	'mercator4v5.0: EC_2.7 transferase transferring phosphorus-containing group & swissprot: Proline-rich receptor-like protein kinase PERK1 & original description: none'
'26.1.4.1.3.2'	'External stimuli response.light.multiple photoreceptor signalling.substrate adaptor module of CUL4-DDB1 ubiquitin ligase complex.regulation of COP1 activity.regulatory factor *(CSU2)'	'ann06064'	'mercator4v5.0: regulatory factor *(CSU2) of COP1 activity & swissprot: Protein COP1 SUPPRESSOR 2 & original description: none'
'35.1'	'not assigned.annotated'	'ann06112'	'mercator4v5.0: not classified & swissprot: GDSL esterase/lipase LIP-4 & original description: none'
'35.1'	'not assigned.annotated'	'ann06107'	'mercator4v5.0: not classified & swissprot: Putative pentatricopeptide repeat-containing protein At1g74580 & original description: none'
'35.1'	'not assigned.annotated'	'ann06101'	'mercator4v5.0: not classified & swissprot: Retrovirus-related Pol polyprotein from transposon RE2 & original description: none'
'35.1'	'not assigned.annotated'	'ann06099'	'mercator4v5.0: not classified & swissprot: Tetraketide alpha-pyrone reductase 2 & original description: none'
'35.1'	'not assigned.annotated'	'ann06095'	'mercator4v5.0: not classified & swissprot: Retrovirus-related Pol polyprotein from transposon TNT 1-94 & original description: none'
'35.1'	'not assigned.annotated'	'ann06092'	'mercator4v5.0: not classified & swissprot: Retrovirus-related Pol polyprotein from transposon RE1 & original description: none'
'35.1'	'not assigned.annotated'	'ann06090'	'mercator4v5.0: not classified & swissprot: Retrovirus-related Pol polyprotein from transposon TNT 1-94 & original description: none'
'35.1'	'not assigned.annotated'	'ann06089'	'mercator4v5.0: not classified & swissprot: Retrovirus-related Pol polyprotein from transposon RE1 & original description: none'
'35.1'	'not assigned.annotated'	'ann06085'	'mercator4v5.0: not classified & swissprot: Putative ribonuclease H protein At1g65750 & original description: none'
'35.1'	'not assigned.annotated'	'ann06084'	'mercator4v5.0: not classified & swissprot: Protein translocase subunit SECA1, chloroplastic & original description: none'
'35.1'	'not assigned.annotated'	'ann06082'	'mercator4v5.0: not classified & swissprot: Protein translocase subunit SecA, chloroplastic & original description: none'
'35.1'	'not assigned.annotated'	'ann06067'	'mercator4v5.0: not classified & swissprot: RNA-directed DNA polymerase homolog & original description: none'
'35.1'	'not assigned.annotated'	'ann06066'	'mercator4v5.0: not classified & swissprot: RNA-directed DNA polymerase homolog & original description: none'
'35.1'	'not assigned.annotated'	'ann06061'	'mercator4v5.0: not classified & swissprot: Retrovirus-related Pol polyprotein from transposon RE1 & original description: none'

'35.1'	'not assigned.annotated'	'ann06059'	'mercator4v5.0: not classified & swissprot: Retrovirus-related Pol polyprotein from transposon RE2 & original description: none'
'35.1'	'not assigned.annotated'	'ann06051'	'mercator4v5.0: not classified & swissprot: Retrovirus-related Pol polyprotein from transposon TNT 1-94 & original description: none'
'35.1'	'not assigned.annotated'	'ann06044'	'mercator4v5.0: not classified & swissprot: Retrovirus-related Pol polyprotein from transposon TNT 1-94 & original description: none'
'35.1'	'not assigned.annotated'	'ann06036'	'mercator4v5.0: not classified & swissprot: Protein trichome birefringence-like 38 & original description: none'
'35.1'	'not assigned.annotated'	'ann06035'	'mercator4v5.0: not classified & swissprot: Protein trichome birefringence-like 38 & original description: none'
'35.1'	'not assigned.annotated'	'ann06034'	'mercator4v5.0: not classified & swissprot: Retrovirus-related Pol polyprotein from transposon RE1 & original description: none'
'35.1'	'not assigned.annotated'	'ann06029'	'mercator4v5.0: not classified & swissprot: Probable inactive purple acid phosphatase 27 & original description: none'
'35.1'	'not assigned.annotated'	'ann06024'	'mercator4v5.0: not classified & swissprot: Cell division control protein 48 homolog C & original description: none'
'35.1'	'not assigned.annotated'	'ann06023'	'mercator4v5.0: not classified & swissprot: Putative ribonuclease H protein At1g65750 & original description: none'
'35.1'	'not assigned.annotated'	'ann06020'	'mercator4v5.0: not classified & swissprot: (S)-coclaurine N-methyltransferase & original description: none'
'35.1'	'not assigned.annotated'	'ann06019'	'mercator4v5.0: not classified & swissprot: (S)-coclaurine N-methyltransferase & original description: none'
'35.1'	'not assigned.annotated'	'ann06014'	'mercator4v5.0: not classified & swissprot: Retrovirus-related Pol polyprotein from transposon RE1 & original description: none'
'35.1'	'not assigned.annotated'	'ann06000'	'mercator4v5.0: not classified & swissprot: Pentatricopeptide repeat-containing protein At4g20740 & original description: none'
'35.1'	'not assigned.annotated'	'ann05999'	'mercator4v5.0: not classified & swissprot: Retrovirus-related Pol polyprotein from transposon TNT 1-94 & original description: none'
'35.1'	'not assigned.annotated'	'ann05998'	'mercator4v5.0: not classified & swissprot: Putative ribonuclease H protein At1g65750 & original description: none'
'35.1'	'not assigned.annotated'	'ann05995'	'mercator4v5.0: not classified & swissprot: Putative ribonuclease H protein At1g65750 & original description: none'
'35.1'	'not assigned.annotated'	'ann05994'	'mercator4v5.0: not classified & swissprot: RNA-directed DNA polymerase homolog & original description: none'
'35.2'	'not assigned.not annotated'	'ann06108'	'mercator4v5.0: not classified & original description: none'
'35.2'	'not assigned.not annotated'	'ann06106'	'mercator4v5.0: not classified & original description: none'
'35.2'	'not assigned.not annotated'	'ann06105'	'mercator4v5.0: not classified & original description: none'
'35.2'	'not assigned.not annotated'	'ann06104'	'mercator4v5.0: not classified & original description: none'
'35.2'	'not assigned.not annotated'	'ann06102'	'mercator4v5.0: not classified & original description: none'
'35.2'	'not assigned.not annotated'	'ann06098'	'mercator4v5.0: not classified & original description: none'
'35.2'	'not assigned.not annotated'	'ann06096'	'mercator4v5.0: not classified & original description: none'
'35.2'	'not assigned.not annotated'	'ann06094'	'mercator4v5.0: not classified & original description: none'
'35.2'	'not assigned.not annotated'	'ann06093'	'mercator4v5.0: not classified & original description: none'
'35.2'	'not assigned.not annotated'	'ann06088'	'mercator4v5.0: not classified & original description: none'
'35.2'	'not assigned.not annotated'	'ann06087'	'mercator4v5.0: not classified & original description: none'
'35.2'	'not assigned.not annotated'	'ann06086'	'mercator4v5.0: not classified & original description: none'
'35.2'	'not assigned.not annotated'	'ann06083'	'mercator4v5.0: not classified & original description: none'
'35.2'	'not assigned.not annotated'	'ann06075'	'mercator4v5.0: not classified & original description: none'
'35.2'	'not assigned.not annotated'	'ann06073'	'mercator4v5.0: not classified & original description: none'
'35.2'	'not assigned.not annotated'	'ann06072'	'mercator4v5.0: not classified & original description: none'
'35.2'	'not assigned.not annotated'	'ann06071'	'mercator4v5.0: not classified & original description: none'
'35.2'	'not assigned.not annotated'	'ann06070'	'mercator4v5.0: not classified & original description: none'
'35.2'	'not assigned.not annotated'	'ann06069'	'mercator4v5.0: not classified & original description: none'
'35.2'	'not assigned.not annotated'	'ann06068'	'mercator4v5.0: not classified & original description: none'
'35.2'	'not assigned.not annotated'	'ann06065'	'mercator4v5.0: not classified & original description: none'

'35.2'	'not assigned.not annotated'	'ann06063'	'mercator4v5.0: not classified & original description: none'
'35.2'	'not assigned.not annotated'	'ann06062'	'mercator4v5.0: not classified & original description: none'
'35.2'	'not assigned.not annotated'	'ann06060'	'mercator4v5.0: not classified & original description: none'
'35.2'	'not assigned.not annotated'	'ann06057'	'mercator4v5.0: not classified & original description: none'
'35.2'	'not assigned.not annotated'	'ann06056'	'mercator4v5.0: not classified & original description: none'
'35.2'	'not assigned.not annotated'	'ann06054'	'mercator4v5.0: not classified & original description: none'
'35.2'	'not assigned.not annotated'	'ann06049'	'mercator4v5.0: not classified & original description: none'
'35.2'	'not assigned.not annotated'	'ann06048'	'mercator4v5.0: not classified & original description: none'
'35.2'	'not assigned.not annotated'	'ann06047'	'mercator4v5.0: not classified & original description: none'
'35.2'	'not assigned.not annotated'	'ann06046'	'mercator4v5.0: not classified & original description: none'
'35.2'	'not assigned.not annotated'	'ann06043'	'mercator4v5.0: not classified & original description: none'
'35.2'	'not assigned.not annotated'	'ann06040'	'mercator4v5.0: not classified & original description: none'
'35.2'	'not assigned.not annotated'	'ann06039'	'mercator4v5.0: not classified & original description: none'
'35.2'	'not assigned.not annotated'	'ann06038'	'mercator4v5.0: not classified & original description: none'
'35.2'	'not assigned.not annotated'	'ann06037'	'mercator4v5.0: not classified & original description: none'
'35.2'	'not assigned.not annotated'	'ann06033'	'mercator4v5.0: not classified & original description: none'
'35.2'	'not assigned.not annotated'	'ann06027'	'mercator4v5.0: not classified & original description: none'
'35.2'	'not assigned.not annotated'	'ann06026'	'mercator4v5.0: not classified & original description: none'
'35.2'	'not assigned.not annotated'	'ann06022'	'mercator4v5.0: not classified & original description: none'
'35.2'	'not assigned.not annotated'	'ann06018'	'mercator4v5.0: not classified & original description: none'
'35.2'	'not assigned.not annotated'	'ann06011'	'mercator4v5.0: not classified & original description: none'
'35.2'	'not assigned.not annotated'	'ann06010'	'mercator4v5.0: not classified & original description: none'
'35.2'	'not assigned.not annotated'	'ann06008'	'mercator4v5.0: not classified & original description: none'
'35.2'	'not assigned.not annotated'	'ann06007'	'mercator4v5.0: not classified & original description: none'
'35.2'	'not assigned.not annotated'	'ann06005'	'mercator4v5.0: not classified & original description: none'
'35.2'	'not assigned.not annotated'	'ann06004'	'mercator4v5.0: not classified & original description: none'
'35.2'	'not assigned.not annotated'	'ann06003'	'mercator4v5.0: not classified & original description: none'
'35.2'	'not assigned.not annotated'	'ann06002'	'mercator4v5.0: not classified & original description: none'
'35.2'	'not assigned.not annotated'	'ann05997'	'mercator4v5.0: not classified & original description: none'
'35.2'	'not assigned.not annotated'	'ann05996'	'mercator4v5.0: not classified & original description: none'
'35.2'	'not assigned.not annotated'	'ann05993'	'mercator4v5.0: not classified & original description: none'
'25.1.5.1'	'Nutrient uptake.nitrogen assimilation.glutamate deamination.glutamate dehydrogenase'	'ann06009'	'mercator4v5.0: glutamate dehydrogenase & swissprot: NADP-specific glutamate dehydrogenase (Fragment) & original description: none'
'25.1.5.1'	'Nutrient uptake.nitrogen assimilation.glutamate deamination.glutamate dehydrogenase'	'ann06006'	'mercator4v5.0: glutamate dehydrogenase & swissprot: NADP-specific glutamate dehydrogenase (Fragment) & original description: none'
'11.2.4.1.1'	'Phytohormone action.auxin.transport.polar auxin transport system.auxin transporter *(PIN)'	'ann06031'	'mercator4v5.0: auxin transporter *(PIN) & swissprot: Auxin efflux carrier component 5 & original description: none'
'19.2.4.1.1'	'Protein homeostasis.ubiquitin-proteasome system.membrane-associated protein degradation.CDC48-NPL4-UFD1 chaperone complex.platform ATPase component *(CDC48)'	'ann06021'	'mercator4v5.0: platform ATPase CDC48 & swissprot: Cell division control protein 48 homolog C & original description: none'
'19.2.3.1.1.9'	'Protein homeostasis.ubiquitin-proteasome system.ubiquitin-fold protein deconjugation.UBQ deconjugation.UBP deubiquitinase activities.deubiquitinase *(UBP24)'	'ann06012'	'mercator4v5.0: deubiquitinase *(UBP24) & swissprot: Ubiquitin carboxyl-terminal hydrolase 24 & original description: none'
'19.2.2.8.2.11'	'Protein homeostasis.ubiquitin-proteasome system.ubiquitin-fold protein conjugation.RING E3 ubiquitin ligase activities.RING-HC-class.E3 ubiquitin ligase *(SR1)'	'ann06030'	'mercator4v5.0: E3 ubiquitin ligase *(SR1) & original description: none'
'18.3.4.3.1'	'Protein modification.lipidation.protein S-acylation.group-C protein S-acyltransferase activities.protein S-acyltransferase *(PAT18)'	'ann06078'	'mercator4v5.0: protein S-acyltransferase *(PAT18) & swissprot: Protein S-acyltransferase 18 & original description: none'

'18.4.21'	'Protein modification.phosphorylation.ABC1 atypical protein kinase'	'ann06074'	'mercator4v5.0: ABC1 atypical protein kinase & swissprot: Protein ABC transporter 1, mitochondrial & original description: none'
'18.4.1.35'	'Protein modification.phosphorylation.TKL protein kinase superfamily.RLCK-VI receptor-like protein kinase'	'ann06015'	'mercator4v5.0: RLCK-VI receptor-like protein kinase & swissprot: Proline-rich receptor-like protein kinase PERK1 & original description: none'
'15.5.19'	'RNA biosynthesis.transcriptional regulation.MADS/AGL-type transcription factor'	'ann06055'	'mercator4v5.0: MADS/AGL-type transcription factor & swissprot: MADS-box transcription factor PHERES 2 & original description: none'
'15.5.19'	'RNA biosynthesis.transcriptional regulation.MADS/AGL-type transcription factor'	'ann06053'	'mercator4v5.0: MADS/AGL-type transcription factor & swissprot: MADS-box transcription factor PHERES 2 & original description: none'
'15.5.37'	'RNA biosynthesis.transcriptional regulation.phosphatase-type DBP-type transcription factor'	'ann06109'	'mercator4v5.0: phosphatase-type DBP-type transcription factor & swissprot: Probable protein phosphatase 2C 49 & original description: none'
'15.5.21'	'RNA biosynthesis.transcriptional regulation.SBP-type transcription factor'	'ann06097'	'mercator4v5.0: SBP-type transcription factor & swissprot: Squamosa promoter-binding-like protein 1 & original description: none'
'16.6.2.1.3'	'RNA processing.organelle machinery.RNA modification.tRNA maturation.uridine 5-carboxymethylaminomethyl modification enzyme *(GidA)'	'ann06017'	'mercator4v5.0: uridine 5-carboxymethylaminomethyl modification enzyme *(GidA) & original description: none'
'16.1.5.1.7'	'RNA processing.pre-RNA splicing.MAC/NTC spliceosome-associated complex.core components.component *(MAC7)'	'ann06041'	'mercator4v5.0: component *(MAC7) of MAC spliceosome-associated complex & swissprot: Regulator of nonsense transcripts 1 homolog & original description: none'
'16.2.1.1.2.3'	'RNA processing.RNA modification.mRNA maturation.RNA 5'-end processing.RNA-dependent RNase P complex.component *(RPP38/POP3)'	'ann06081'	'mercator4v5.0: component *(RPP38/POP3) of RNA-dependent RNase P complex & original description: none'
'16.2.6.2.7.3'	'RNA processing.RNA modification.RNA methylation.tRNA methylation.TRM7-TRM732/TRM734 tRNA cytidine/guanine-methyltransferase complex.component *(TRM734)'	'ann06100'	'mercator4v5.0: component *(TRM734) of TRM7-TRM732/TRM734 tRNA cytidine/guanosine-methyltransferase complex & original description: none'
'16.2.6.2.18'	'RNA processing.RNA modification.RNA methylation.tRNA methylation.MnmG-type 5,10-methylene-THF-dependent tRNA uridine methyltransferase'	'ann06017'	'mercator4v5.0: MnmG-type 5,10-methylene-THF-dependent tRNA uridine methyltransferase & original description: none'
'24.2.3.6'	'Solute transport.carrier-mediated transport.APC superfamily.borate transporter *(BOR)'	'ann06111'	'mercator4v5.0: borate transporter *(BOR) & swissprot: Probable boron transporter 2 & original description: none'
'24.2.5.2.1'	'Solute transport.carrier-mediated transport.BART superfamily.AEC family.auxin efflux transporter *(PIN)'	'ann06031'	'mercator4v5.0: auxin efflux transporter *(PIN) & swissprot: Auxin efflux carrier component 5 & original description: none'
'24.3.7'	'Solute transport.channels.calcium-permeable channel *(OSCA)'	'ann06042'	'mercator4v5.0: calcium-permeable channel *(OSCA) & swissprot: CSC1-like protein At3g54510 & original description: none'
'22.4.2.3'	'Vesicle trafficking.exocytic trafficking.exocytosis regulation.regulatory protein *(NERD1)'	'ann06032'	'mercator4v5.0: regulatory protein *(NERD1) of exocytic trafficking & original description: none'
'14.1.2.4'	DNA damage response.DNA damage sensing and signalling.ATR pathway.checkpoint activation factor *(MEI1)'	'ann06013'	'mercator4v5.0: DNA damage checkpoint activation factor *(MEI1) & original description: none'
'50.1.3'	Enzyme classification.EC_1 oxidoreductases.EC_1.3 oxidoreductase acting on CH-CH group of donor'	'ann06077'	'mercator4v5.0: EC_1.3 oxidoreductase acting on CH-CH group of donor & swissprot: Protoporphyrinogen oxidase, chloroplastic & original description: none'
'50.3.2'	Enzyme classification.EC_3 hydrolases.EC_3.2 glycosylase'	'ann06110'	mercator4v5.0: EC_3.2 glycosylase & swissprot: Polygalacturonase At1g48100 & original description: none'
BINCODE	NAME	IDENTIFIER	DESCRIPTION
'18.4.24.2.7'	Protein modification.phosphorylation.protein serine/threonine phosphatase superfamily.PPM/PP2C Mn/Mg-dependent phosphatase families.clade G phosphatase *(DBP)'	'ann06109'	'mercator4v5.0: clade G phosphatase *(DBP) & swissprot: Probable protein phosphatase 2C 49 & original description: none'
'15.5.19'	RNA biosynthesis.transcriptional regulation.MADS/AGL-type transcription factor'	'ann06052'	mercator4v5.0: MADS/AGL-type transcription factor & swissprot: MADS-box transcription factor PHERES 2 & original description: none'
'15.5.19'	RNA biosynthesis.transcriptional regulation.MADS/AGL-type transcription factor'	'ann06050'	'mercator4v5.0: MADS/AGL-type transcription factor & swissprot: MADS-box transcription factor PHERES 2 & original description: none'
