# Supplementary figures and images for: The chromosome-scale genome and the genetic resistance machinery against insect herbivores of the Mexican toloache, Datura stramonium
Source: G3 (Bethesda). 2023 Dec 19;14(2):jkad288. doi: 10.1093/g3journal/jkad288 (PMC10849327; doi:10.1093/g3journal/jkad288)

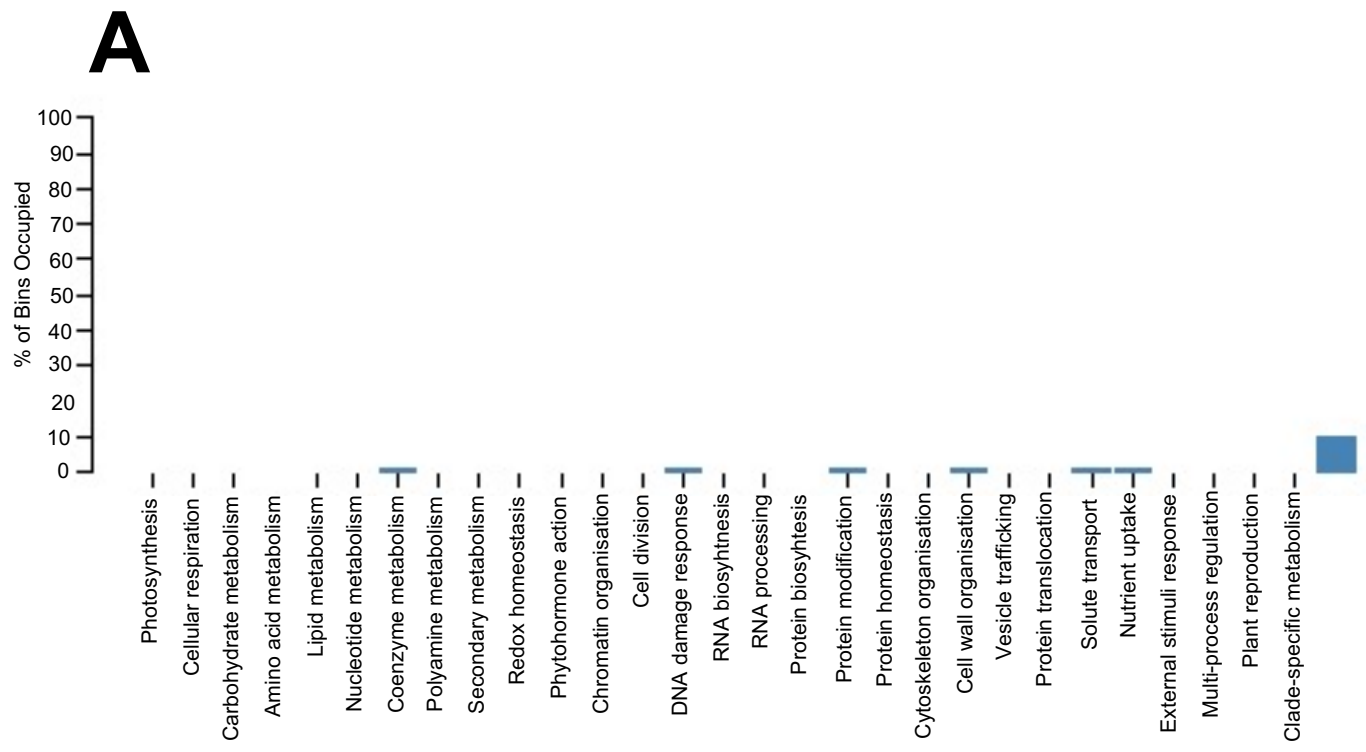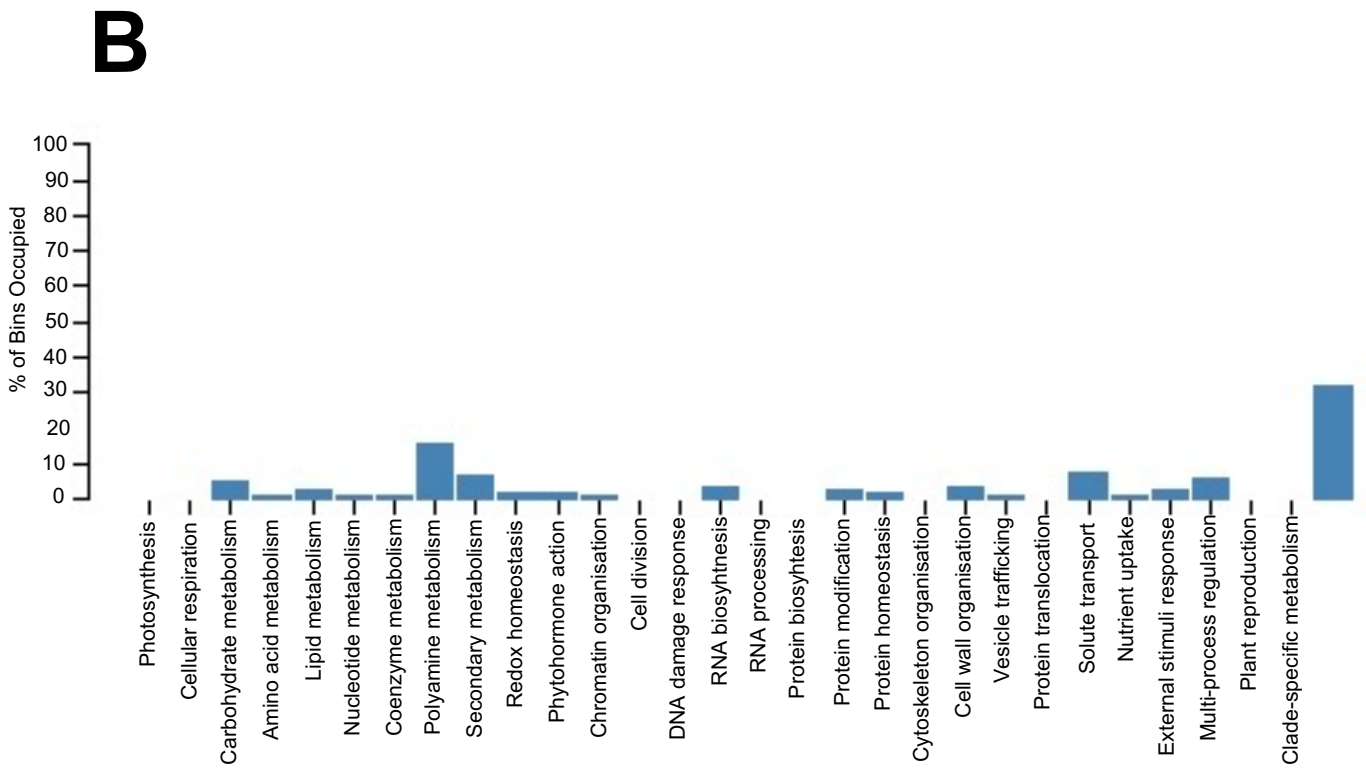

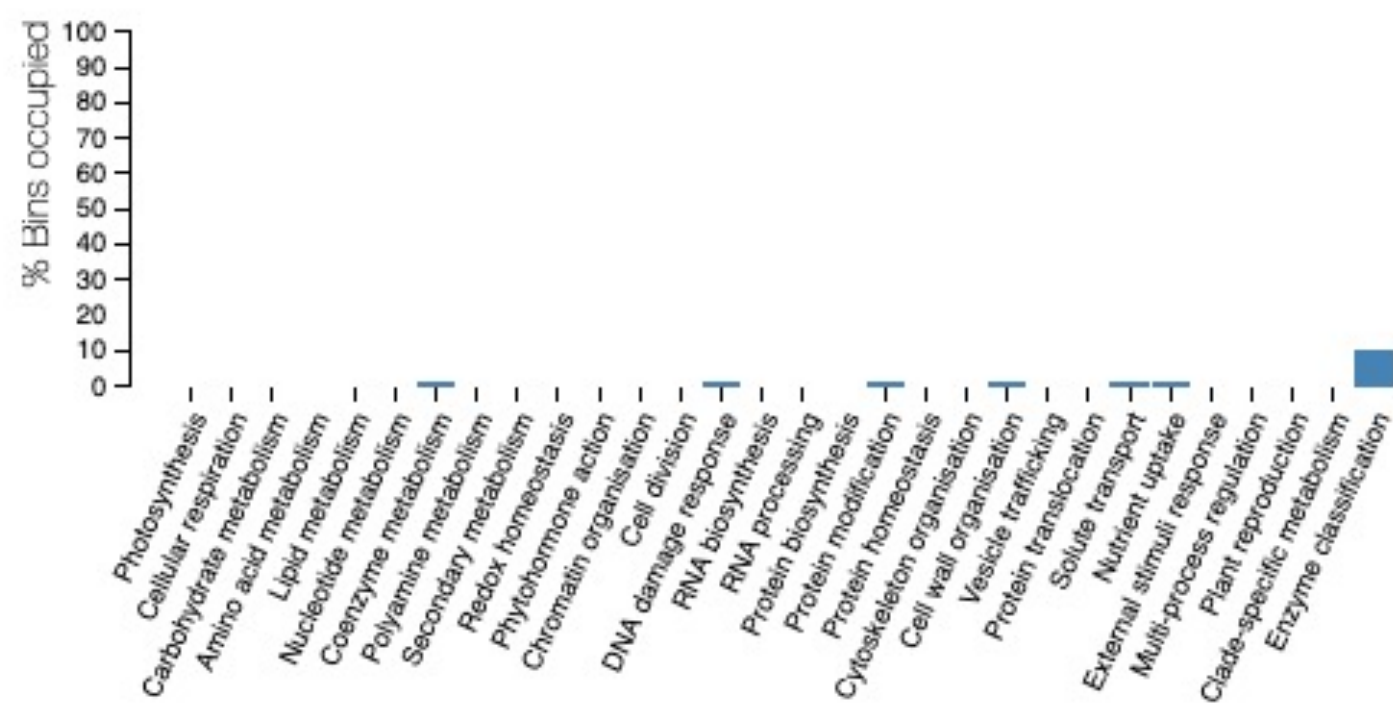

Supplement: jkad288_Supplementary_Data [file jkad288_supplementary_data.zip › Figure_S1_G3-2023-404717.pdf]
